# DNA Damage Response and Repair Gene Alterations Increase Tumor Mutational Burden and Promote Poor Prognosis of Advanced Lung Cancer

**DOI:** 10.3389/fonc.2021.708294

**Published:** 2021-09-15

**Authors:** Jiawei Dai, Minlin Jiang, Kan He, Hao Wang, Peixin Chen, Haoyue Guo, Wencheng Zhao, Hui Lu, Yayi He, Caicun Zhou

**Affiliations:** ^1^SJTU-Yale Joint Center for Biostatistics and Data Science, Department of Bioinformatics and Biostatistics, School of Life Sciences and Biotechnology, Shanghai Jiao Tong University, Shanghai, China; ^2^Department of Medical Oncology, Shanghai Pulmonary Hospital, Tongji University Medical School Cancer Institute, Tongji University School of Medicine, Shanghai, China; ^3^Medical School, Tongji University, Shanghai, China

**Keywords:** DNA damage and repair, DDR, tumor mutational burden, tumor microenvironment, immune infiltration, heterogeneity, prognosis, lung cancer

## Abstract

DNA damage response and repair (DDR) gene alterations increase tumor-infiltrating lymphocytes, genomic instability, and tumor mutational burden (TMB). Whether DDR-related alterations relate to therapeutic response and prognosis in lung cancer lacking oncogenic drivers remains unknown. Pretherapeutic cancer samples of 122 patients [86 non-small cell lung cancer and 36 small cell lung cancer (SCLC)] harboring no *EGFR/ALK* alterations were collected. Through whole-exome sequencing, we outlined DDR mutational landscape and determined relationships between DDR gene alterations and TMB or intratumoral heterogeneity. Then, we evaluated the impacts of DDR gene alterations on therapeutic response and prognosis and established a DDR-based model for prognosis prediction. In addition, we investigated somatic interactions of DDR genes and immunomodulatory genes, immune expression patterns, immune microenvironment, and immune infiltration characteristics between DDR-deficient and DDR-proficient samples. Samples from cBioportal datasets were utilized for verification. We found that deleterious DDR gene alterations were closely associated with higher TMB than proficient-types (*p* < 0.001). DDR mechanisms attach great importance to the determination of patients’ prognosis after chemotherapy, and alterations of base excision repair pathway in adenocarcinoma, nucleotide excision repair in squamous carcinoma, and homologous recombination pathway in SCLC tend to associate with worse progression-free survival to first-line chemotherapy (all *p* < 0.05). A predictive nomogram model was constructed incorporating DDR-related alterations, clinical stage, and smoking status, with the area under curve values of 0.692–0.789 for 1- and 2-year receiver operating characteristic curves in training and testing cohorts. Furthermore, DDR-altered tumors contained enhanced frequencies of alterations in various genes of human leukocyte antigen (HLA) class I pathway including *TAP1* and *TAP2* than DDR-proficient samples. DDR-deficient types had lower expressions of STING1 (*p* = 0.01), CD28 (*p* = 0.020), HLA-DRB6 (*p* = 0.014) in adenocarcinoma, lower TNFRSF4 (*p* = 0.017), and TGFB1 expressions (*p* = 0.033) in squamous carcinoma, and higher CD40 (*p* = 0.012) and TNFRSF14 expressions *(p* = 0.022) in SCLC. DDR alteration enhanced activated mast cells in adenocarcinoma (*p* = 0.044) and M2 macrophage in squamous carcinoma (*p* = 0.004) than DDR-proficient types. Collectively, DDR gene alterations in lung cancer without oncogenic drivers are positively associated with high TMB. Specific DDR gene alterations tend to associate with worse progression-free survival to initial chemotherapy.

## Introduction

Driver gene mutations are important for advanced non-small cell lung cancer (NSCLC) to develop and several targets often drive neoplastic transformation (1–3). Tyrosine kinase inhibitors (TKIs) can treat patients with mutations of driver genes such as epidermal growth factor receptor (*EGFR*) and anaplastic lymphoma kinase (*ALK*) and act as the first-line therapy for them (4, 5). However, many NSCLC patients lacking oncogenic drivers respond only modestly to targeted therapies (6). At present, chemotherapy remains an important therapeutic scheme in these patients (7). Immunotherapy for these patients might become a promising strategy (8, 9). Next-generation sequencing (NGS) technology characterizes genomic alterations and demonstrated the association of tumor mutation burden (TMB) with immune checkpoint inhibitors (10, 11). Other genomic signatures have also been found important for predicting the efficacy of targeted agents (12). These suggest the potential for examining efficacy predictors for patients’ prognosis through genetic profiling.

Genes in DNA damage response and repair (DDR) system are crucial for maintaining genome stability. Impaired DDR function is a key determinant of tumor development and therapeutic outcomes conversely (13). Based on mechanistic, biochemical, and genetic criteria, functional pathways were defined including diverse DDR genes. Proteins of the same pathway can work synergistically to repair specific DDR damage (14, 15). The base excision repair (BER) and nucleotide excision repair (NER) pathways mainly correct damage of DNA base. Mismatch repair (MMR) repairs base mispairs as well as small loops that often appear in repetitive DNA sequences. Non-homologous end joining (NHEJ), homologous recombination (HR), and Fanconi anemia (FA) pathways are responsible for repairing DNA strand breaks and complex events such as interstrand crosslinks (16). Hypothetically, given the dysfunction of restoring chemotherapy-induced DNA damage, DDR-damaged neoplasms appear more sensitive to platinum-based chemotherapy, which has been verified in cancers such as ovarian cancer, triple negative breast cancer, and urothelial carcinoma (17–20). With the development of immunotherapy, DDR pathways have been reemphasized, and their alterations are closely associated with genetic characteristics like high TMB *via* accumulation of some uncorrected DNA damage.

Few reports identified the genomic landscape and transcriptomic characteristics of DNA damage response deficiency in lung cancer patients lacking TKI-related oncogenic drivers. Here, we first investigated the mutational profiles in *EGFR*−/*ALK*− lung cancer patients and the associations of DDR gene alterations with TMB and intratumor heterogeneity (ITH). Then, patients’ prognoses including overall survival (OS) and progression-free survival (PFS) were evaluated in distinct DDR pathways and pathological subtypes. Furthermore, we also investigated somatic interactions of DDR-genes and immunomodulatory genes, immune expression patterns, and immune microenvironment and immune infiltration characteristics between DDR-deficient and DDR-proficient samples. Based on these above analyses, we demonstrated the important role of specific DDR gene alterations in therapeutic response and indicated the promising use of immunotherapy in DDR-altered patients without *EGFR/ALK* mutations.

## Methods and Materials

### Ethical Approval

All study plans and experimental protocols were submitted to the ethics/licensing committee of Shanghai Pulmonary Hospital, and all of them had been approved. Written informed consents were obtained from all patients involved. All methods, personal training, and experiments were performed following relevant regulations and guidelines.

### Study Design, Participants, and Sample Collection

This study aimed to enroll lung cancer patients without *EGFR* [single-nucleotide variant (SNV), insertion/deletion (INDEL)] or *ALK* (fusion) alterations and analyze their DDR-related genomic and transcriptomic characteristics. All patients enrolled received first-line chemotherapy. All eligible patients received polymerase chain reaction (PCR) assays for *EGFR* and *ALK* before any therapy. After that, samples from 122 qualifying advanced lung cancer patients were eligible, including 86 NSCLC and 36 SCLC participants (**Table S1**). Tumor specimens and blood samples were collected as we previously described (21).

### Sample Preparation and Tumor Sequencing

Sample storage, DNA extraction, and DNA sequencing were done as previously described (21).

Somatic variation was detected by Genome Analysis Toolkit (GATK, Version 4.1.7.0). We followed the workflows of Burrows–Wheeler aligner (BWA, Version 0.7.17-r1198) for aligning sequencing data to the hg19 genome (GRch37). Duplicated reads underwent subsequent marking and were removed by the GATK Picard tool. Base quality score was recalibrated *via* BaseRecalibrator and ApplyBQSR functions of GATK, and Mutect2 from GATK was designed for calling SNVs and INDELs from tumor-normal matched pairs. Above analyses were performed on the cloud-based genomic analysis platform: Biomedical Data Analysis Platform (BMAP, https://bmap.sjtu.edu.cn/). Significantly mutated genes mutated more frequently than expected accidentally were determined through MutSigCV (Version: 1.41) with q values <0.1 (22) in our study.

### Examination of *EGFR* Mutation and *ALK* Rearrangement

Tissue DNA or RNA was extracted based on the manufacturer’s protocol, and reversed transcript would be performed for extracted RNA for subsequent PCR amplification. *EGFR* mutations and *EML4-ALK* fusion were detected as described (21).

### Gene Sets and Genes of DDR Pathways Selected, Pathogenicity Assessment, and Deleterious Mutation Determination

We evaluated seven major DDR pathways, BER, MMR, HR, NER, NHEJ, FA, and cell cycle checkpoint in our study. A total of 74 DDR genes were assembled as being associated with DDR, grouped into different functional pathways from published resources (15, 19, 23–27) (**Table S2**). We considered all loss-of-function alterations deleterious, including nonsense mutations, splice site, or frameshift alterations (**Table S3**) (19). Two diverse methods were applied to determine the functional impacts of missense mutations: (1) by *in silico* functional analysis, all missense mutations that were classified as “probably damaging” or “possibly damaging” in Polyphen2 (28) or “high” or “medium” in MutationAssessor (29) were recognized deleterious; (2) we manually reviewed the missense mutations in Catalogue of Somatic Mutations in Cancer (COSMIC) (30), algorithmically identified recurrent hotspot mutations (31), and annotated oncogenicity *via* OncoKB. DDR gene alterations were defined as deleterious DDR mutations. In our study, DDR-deficient subtype was defined as individuals with deleterious DDR mutations. DDR-proficient subtype was defined as individuals without deleterious DDR mutations. The deficient and proficient individuals in a particular pathway were identified as individuals with or without mutation in this particular pathway.

### Mutational Signature and Cluster Analysis

We used SignatureAnalyzer to infer mutational signatures of our samples (32, 33) (http://software.broadinstitute.org/cancer/cga/msp). SignatureAnalyzer applied a Bayesian variant of non-negative matrix factorization algorithm for signature analysis, and mutational signatures were identified by comparing with 30 COSMIC mutational signatures.

Consensus clustering was performed using the unsupervised tool named ConsensusClusterPlus (34). We set Pearson correlation distances for distance, 80% item resampling for pItem, and 10 resamplings for reps. Eventually, we determined three clusters among these patients.

### Calculation of Somatic TMB and MATH Scores

We calculated TMB by dividing the total number of cancer tissue non-synonymous variations (SNV and INDEL, allele frequency >5%) by the length of the whole-exome sequencing (WES) panel. In addition, we used the MATH score as a quantitative measure for ITH, which considered the width of variant allele frequency distribution for calculation (35).

### Expression Level of Immune-Related Genes

To figure out relevant expression levels of immune-related genes in lung cancer patients without *EGFR/ALK* mutations between DDR-deficient and DDR-proficient groups, appropriate data were obtained from databases like The Cancer Genome Atlas (TCGA) obtained from cBioportal, lung adenocarcinoma (LADC) cohort from dataset “Lung Adenocarcinoma (TCGA, Firehose Legacy)” (https://www.cbioportal.org/study/summary?id=luad_tcga), lung squamous cell carcinoma (LUSC) cohort from dataset “Lung Squamous Cell Carcinoma (TCGA, Firehose Legacy)” (https://www.cbioportal.org/study/summary?id=lusc_tcga), and small cell lung cancer (SCLC) cohort from dataset “Small Cell Lung Cancer (U Cologne, Nature 2015)” (https://www.cbioportal.org/study/summary?id=sclc_ucologne_2015). After excluding patients with *EGFR/ALK* genomic alterations, a total of 64, 72, and 116 patients were included in the LADC, LUSC, and SCLC cohorts, respectively.

### Nomogram Model Construction

We performed univariate and multivariate Cox regression analyses considering clinical information (including age, gender, smoking history, clinical stage, and pathological type), and TMB, ITH, and DDR gene mutations as variables. We divided patients into training and testing groups randomly (7:3). Factors selected from Cox regression were included for building a nomogram model in the training group (36). Prognostic values at 1- and 2-year survival of lung cancer patients were predicted. Then, time-dependent receiver operating characteristic (ROC) curves were performed to evaluate the performance of this nomogram in both training and testing datasets. After that, Kaplan–Meier survival analysis was further used for evaluating the clinical value of this predictive model. The cutoff value of the total point of nomogram was determined using R package “survminer.”

### External Datasets for Verification

The findings of our data were validated using external datasets. We used datasets from cBioportal to form the external validation cohort for verifying the performance of the clinical prediction model based on the DDR mutational status. The validation cohorts we used for exploring the relationships of DDR gene alterations with TMB, ITH, and survival, and verifying model performance were obtained from cBioportal (LADC, LUSC, and SCLC cohorts mentioned above). Patients with *EGFR/ALK* genomic alterations were excluded.

### Clinical Outcomes and Statistical Methods

We determined objective response rate (ORR), disease control rate (DCR), PFS, and OS based on Response Evaluation Criteria in Solid Tumors version 1.1 (37). PFS was defined as the interval from the start of first-line therapy to the date of disease progression or death. OS was defined as the interval from the start of first-line therapy to death. Wilcoxon rank-sum test was applied for comparing mutational burden between defined subgroups, and two-tailed Student’s test was performed for comparing immune-related gene expression and immune infiltration between DDR-deficient and DDR-proficient subtypes. Associations between DDR-deficient and DDR-proficient groups were analyzed by chi^2^ or Fisher’s exact tests for qualitative data. We used maftools (38) for the detection of co-occurring or mutually exclusive sets of genes, in which pairwise Fisher’s exact test was used to explore significant gene pairs. Multiple logistic regression analysis was performed by categorizing TMB based on the median to analyze the effects of DDR mutations and smoking on TMB. The Kaplan–Meier curves with log-rank test were performed for testing survival differences between two subgroups. Cox regression was also used for the determination of clinical values of single DDR genes. We also used Benjamini–Hochberg test for *p*-value correction to test multiple hypotheses when appropriate. Extern mutational and clinical data were obtained from TCGA *via* cBioportal. All visualizations were achieved using R software. We defined “*” as statistically significant (*p* < 0.05), “**” as highly statistically significant (*p* < 0.01), and “***” as very highly statistically significant (*p* < 0.001). We defined *p* < 0.05 as statistically significant.

## Results

### DDR Mutational Landscape of Advanced Lung Cancers Without *EGFR* or *ALK* Oncogenic Driver Alterations

**Figure 1:**
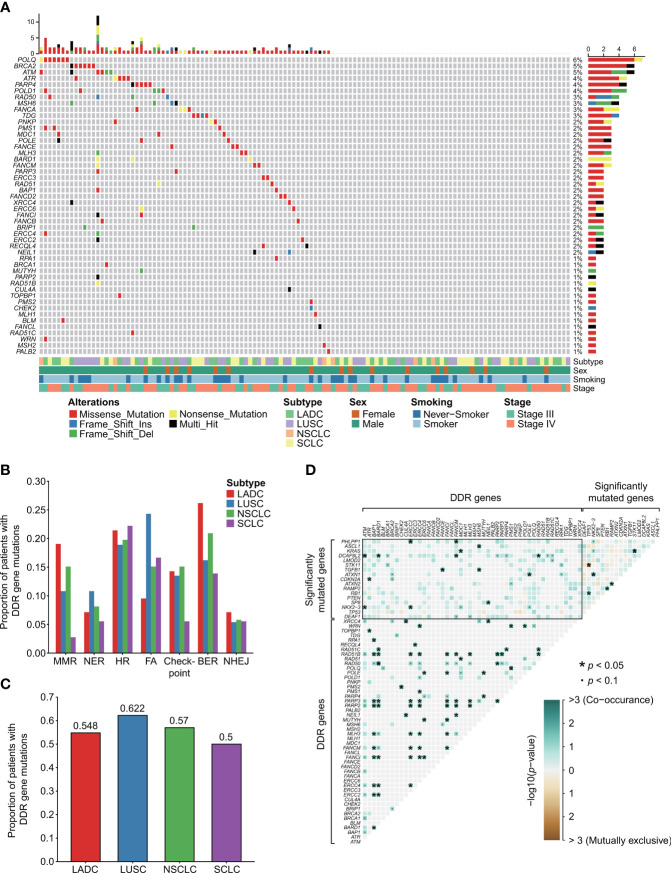
DDR mutational landscape of advanced lung cancers without *EGFR* or *ALK* oncogenic driver alterations. **(A)** Clinical features and DDR mutation spectrum of 122 advanced lung cancer samples without *EGFR* or *ALK* mutations/translocations. A total of 67 patients were identified with deleterious alterations of DDR genes. **(B)** The number and proportion of mutation samples that occurred in the seven types of DDR related pathways. Chi-square or Fisher’s exact tests were used to compare the mutation frequency between distinct subtypes of lung cancers. **(C)** The proportion of samples with mutations in all DDR genes. **(D)** Somatic interactions detection. There were widespread comutations between DDR genes and significantly mutated genes. No mutually exclusive sets between DDR genes and significantly mutated genes were found. Unlike significantly mutated genes showing both comutations and mutual exclusions, between DDR genes extensive comutations were extremely frequent. DDR gene coalterations occur both in the same and different DDR pathways. We used the R package “maftools” for the detection of co-occurring or mutually exclusive sets of genes. “*” means *p* < 0.05. “•” means *p* < 0.1. The “checkpoint” referred to “cell cycle checkpoint”.

We identified the clinicopathological features and DDR mutation spectrum of 122 advanced lung cancer samples without *EGFR* or *ALK* mutations/translocations. In brief, 86.9% (106/122) of patients were male, and 69.7% (85/122) of patients had smoking history (**Figure 1A**). In our study, the proportion of pathologically determined LADC and SCLC were 34.4% (42/122) and 29.5% (36/122), respectively. LUSC accounted for 30.3% (37/122) (**Figure 1A**). A total of 67 patients were identified with deleterious alterations of DDR genes (67/122, 54.9%, **Figure 1A**; **Table S3**). Their clinical characteristics are summarized in **Table S1**. *POLQ*, *BRCA2*, *ATM*, *ATR*, *PARP4*, and *POLD1* alterations were most commonly observed in the entire advanced lung cancer cohort (**Figure 1A**). Different histopathological types exhibited specific mutation characteristics in the DDR pathways (**Figure 1B**). For example, MMR alterations were relatively more common in LADC (8/42, 19.0%) but less frequent in SCLC (1/36, 2.8%; LADC vs. SCLC, *p* = 0.059). FA alterations were observed more frequently in LUSC (9/37, 24.3%), while 9.5% were observed in the LADC subtype (4/42, 9.5%, *p* = 0.077). However, the proportion of patients with DDR gene alterations revealed no difference either between NSCLC and SCLC or between LADC and LUSC (**Figure 1C**).

Then, we explored the relationship between distinct clinical characteristics and DDR mutation. No statistical difference was observed in smoking (*p* = 0.899) and gender (*p* = 0.335) between DDR-deficient and DDR-proficient subtypes by chi^2^ or Fisher’s exact test. The same result was also observed in age by Student’s t-test (*p* = 0.832). For SCLC, a statistically significant result was observed between smoking and DDR mutation (*p* = 0.045). No significant relationship was detected between these clinical factors and DDR mutation in LADC and LUSC (all *p* > 0.05).

By detecting somatic interactions of genes of our data, we found that there were widespread comutations between DDR genes and significantly mutated genes. For example, significantly mutated gene *KRAS* alteration was observed co-occurring with alterations of DDR genes *MDC1* (*p* = 0.026); *DCAF8L2* alteration was observed co-occurring with alterations of *ATM* (*p* = 0.010), *BARD1* (*p* = 0.022), *FANCM* (*p* = 0.022), and *RAD50* (*p* = 0.042, **Figure 1D**). No mutually exclusive sets between DDR genes and significantly mutated genes were found. Unlike significantly mutated genes showing both comutations and mutual exclusions, between DDR genes, extensive comutations were extremely frequent. DDR gene coalterations occur both in the same and different DDR pathways. For instance, HR gene *RAD51B* was observed comutated with *BAP1* (*p* = 0.017), *BARD1* (*p* = 0.025), *RAD50* (*p* = 0.033) in the same pathway, and *ERCC2* (*p* = 0.017), *ERCC4* (*p* = 0.017), *FANCI* (*p* = 0.017), *FANCM* (*p* = 0.025), *MLH3* (*p* = 0.025), *PARP2* (*p* = 0.008), and *RARP3* (*p* = 0.017) in other DDR pathways.

### DDR-Altered Malignancies Contained Increased Mutational Load

**Figure 2:**
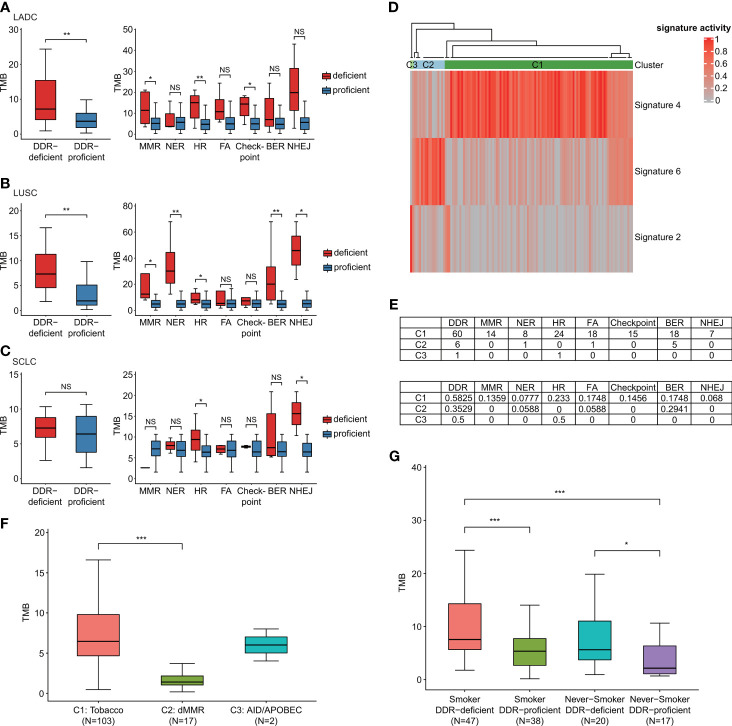
DDR-altered malignancies contained increased mutational load. **(A)** The effect of DDR gene alterations on LADC patients’ TMB levels (own dataset, n = 42). **(B)** The effect of DDR gene alterations on LUSC patients’ TMB levels (own dataset, n = 37). **(C)** The effect of DDR gene alterations on SCLC patients’ TMB levels (own dataset, n = 36). When comparing the TMB status of groups with DDR gene alterations to those harboring no DDR-related genomic mutations, TMB was comparably higher in LADC and LUSC participants with DDR genomic alteration than DDR-proficient patients. SCLC also showed a similar trend although no significant difference was observed. **(D)** Three mutational signatures (signature 2, 4, and 6) and three subgroups (C1–C3) *via* unsupervised clustering analysis. **(E)** The mutation number and proportion of different pathways in different molecular subgroups. **(F)** TMB distribution of different molecular subgroups C1–C3. Samples most relevant to signature 6 (dMMR) failed to show relatively high TMB. **(G)** Effects of DDR gene mutations and smoking on TMB. Wilcoxon rank-sum test was utilized to study the relationship of DDR gene alterations with TMB. *p < 0.05, **p < 0.01, and ***p < 0.001. The “checkpoint” referred to “cell cycle checkpoint”. The "NS" referred to "not significant".

In our study, the median TMB for all patients enrolled was 6.06 mutations/megabase, ranged from 0.17 to 67.95. When comparing the TMB status of groups with DDR gene alterations to those harboring no DDR-related genomic mutations, we identified that TMB was comparably higher in participants with DDR genomic alteration than DDR-proficient patients (*p* = 0.007 in LADC cohort and *p* = 0.003 in LUSC cohort; **Figure 2**). SCLC also showed a similar trend, although no significant difference was observed (*p* = 0.389). Different histological subtypes revealed substantial differences in TMB distribution in different DDR mutation states. For LADC, groups with mutations of MMR (*p* = 0.039), HR (*p* = 0.006), and cell cycle checkpoint genes (*p* = 0.017) showed significantly higher TMB than corresponding proficient groups, while NER (*p* = 0.922), FA (*p* = 0.075), BER (*p* = 0.188), and NHEJ (*p* = 0.171) failed to reveal the difference (**Figure 2A**). LUSC patients with DDR deficiencies of MMR (*p* = 0.012), NER (*p* = 0.002), HR (*p* = 0.044), BER (*p* = 0.009), and NHEJ pathways (*p* = 0.024) showed significantly higher TMB than corresponding proficient participants (**Figure 2B**). However, for SCLC, only HR (*p* = 0.044) and NHEJ (*p* = 0.013) alterations showed significant differences; MMR (*p* = 0.222), NER (*p* = 0.622), FA (*p* = 0.467), BER (*p* = 0.396), and cell cycle checkpoint alterations (*p* = 0.622) showed no statistical significance (**Figure 2C**). Then, we classified high- and low-TMB using a cutoff of median TMB value (**Table S4**). By binary comparison, positive associations between TMB-high group and genomic alterations were observed in the mutational status of DDR (*p* = 0.006), HR (*p* = 0.014), and cell cycle checkpoints (*p* = 0.013).

We also identified three mutational signatures (signatures 2, 4, and 6) compared with 30 COSMIC mutational signatures and divided the whole cohort into three subgroups (C1–C3) *via* unsupervised clustering analysis latter. **Figure 2D** shows that C2 subgroup had enriched signature 6, which might have a relationship with deficient MMR (dMMR). High proportions of DDR gene mutations of samples were detected in groups C1 and C2 (**Figure 2E**). By comparing the proportions of samples carrying various DDR gene mutations in each signature/cluster group, we found that C1 group had the highest proportion of MMR alterations (13.59%) in comparison with C2 and C3 groups. Samples most relevant to signature 6 (dMMR) failed to show relatively high TMB (**Figure 2F**). These suggested, although the signature 6 mutation feature was detected in our data, the effects of signature 6 and mutational status of DDR genes on TMB were different. The mutational status of DDR genes reflected the mutation load of lung cancer patients better. We then compared the effects of DDR mutations and smoking on TMB (**Figure 2G**). By Wilcoxon rank-sum test, DDR-deficient subtype was associated with significantly higher TMB among both smokers and non-smokers. No significant correlation was observed between smoking status and TMB among both DDR-deficient and DDR-proficient cohorts. We also performed multiple logistic regression analysis by categorizing TMB based on the median to analyze the effects of DDR mutation and smoking on TMB. It showed that DDR mutations and smoking were both significantly associated with increased TMB (*p* = 0.006 and *p* = 0.030, respectively). The regression coefficient of DDR mutation (1.05) was much larger than smoking (0.92), which suggested that in lung cancer patients without *EGFR/ALK* alterations, DDR mutation had a greater impact on TMB than smoking.

**Figure 3:**
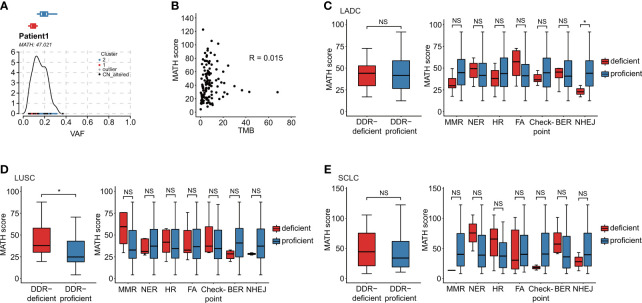
Association of DDR gene alterations with ITH. **(A)** MATH score was calculated for the evaluation of ITH diversity. Take Patient 1 as an example. **(B)** TMB is a quantification of tumor mutations, and MATH score is a quantification of the ITH diversity. In our data, the patient’s Pearson correlation was only 0.015, which negated the correlation between TMB and ITH. **(C–E)** Relationship between MATH score and DDR alteration in distinct histological subtypes: LADC (n = 42), LUSC (n = 37), and SCLC (n = 36). **(C)** Relationship between MATH score and distinct alterations of DDR pathways in LADC subtype. **(D)** Relationship between MATH score and distinct alterations of DDR pathways in LUSC subtype. **(E)** Relationship between MATH score and distinct alterations of DDR pathways in SCLC subtype. Wilcoxon rank-sum test was utilized to study the relationship of DDR gene alterations with ITH. *p < 0.05. The "NS" referred to "not significant". The “checkpoint” referred to “cell cycle checkpoint”.

MATH score was calculated for ITH evaluation (**Figure 3A**). In our study, Pearson correlation was only 0.015 for TMB and ITH (*p* = 0.873), which negated the correlation between TMB and ITH (**Figure 3B**). DDR-deficient patients showed significantly higher ITH than DDR-proficient patients (*p* = 0.043) in LUSC, while no significance was suggested in LADC or SCLC (**Figures 3C–E**). For specific DDR pathways, significant difference was only observed in LADC patients with NHEJ alterations (*p* = 0.048; **Figure 3C**). In SCLC, DDR gene alterations had no significant effect on ITH (**Figure 3E**). Among all patients, except for NHEJ alteration (*p* = 0.036), mutations in the other DDR pathways were not related to MATH score. By dividing all patients into high and low MATH score group using a cutoff of median value, we found no correlation between ITH status and DDR pathway alterations (*p* > 0.05, **Table S5**).

Based on TMB and ITH, survival analyses were performed to identify their predictive and prognostic value for first-line chemotherapy in lung cancer patients without *EGFR/ALK* mutations. High-TMB patients suggested no survival benefits in both PFS and OS compared with TMB-low patients in all (*p* = 0.66, *p* = 0.46), NSCLC (*p* = 0.43, *p* = 0.46), LADC (*p* = 0.51, *p* = 0.92), LUSC (*p* = 0.26, *p* = 0.43), and SCLC cohorts (*p* = 0.2, *p* = 0.78; **Figures S1A, S1B****)**. We also observed no significant prognosis value of ITH in either NSCLC or SCLC (**Figures S1C, S1D****)**.

### Evaluating Therapeutic Response and Prognosis by DDR Pathway and Single Genes

The relationship between DDR pathways and therapeutic response to initial chemotherapy in distinct populations was identified. **Table S6** suggested no significant correlations among DDR gene alterations with regards to ORR and DCR.

**Figure 4:**
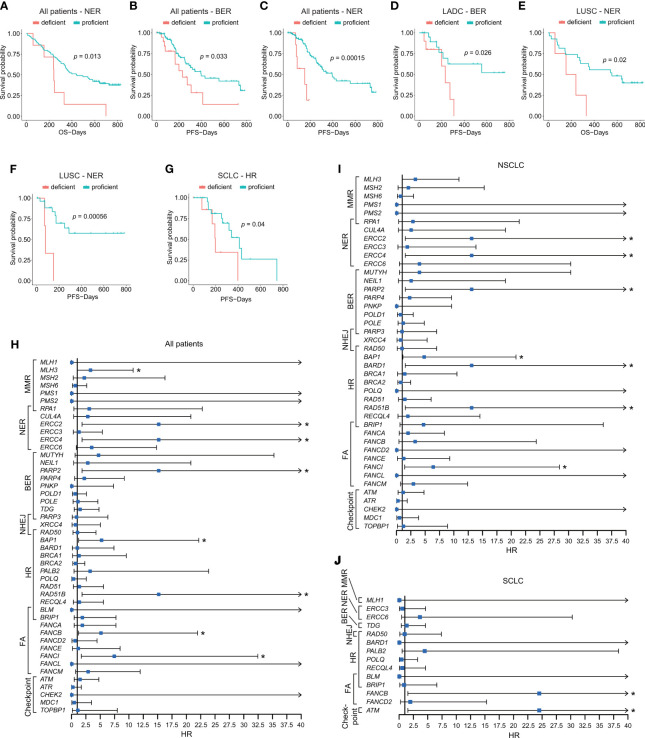
Impacts of alterations of DDR pathways and single DDR genes on patients’ prognosis. **(A–C)** Survival analyses of DDR gene alterations on PFS and OS in NER and BER pathways in all patients. **(D)** Survival analysis of BER alteration on PFS in LADC cohort. **(E)** Survival analysis of NER alteration on OS in LUSC cohort. **(F)** Survival analysis of NER alteration on PFS in LUSC cohort. **(G)** Survival analysis of HR alteration on PFS in SCLC cohort. **(H)** Impacts of somatic alterations in single-gene level on OS in all patients. **(I)** Impacts of somatic alterations in single-gene level on OS in NSCLC patients. **(J)** Impacts of somatic alterations in single-gene level on OS in SCLC patients. The Kaplan–Meier and Cox regression analysis were performed. *p < 0.05. The “checkpoint” referred to “cell cycle checkpoint.”.

To assess the predictive role of DDR in first-line chemotherapy, we identified PFS and OS by analyzing different gene sets and individual DDR genes. We first compared patients’ survival between DDR-deficient and DDR-proficient patients; however, no clinical association was found when analyzing PFS and OS in distinct cohorts (*p* > 0.05; **Figure S2**). We then evaluated the clinical efficacy based on levels of diverse DDR pathways. Among all patients, NER aberrations showed poor PFS (vs. NER-proficient patients, 151.000 days, 95% CI 13.569–288.431 vs. 394.000 days, 95% CI 275.953–512.047; *p* < 0.001) and OS (vs. NER-proficient patients, 241.000 days, 95% CI 235.868–246.132 vs. 438.000 days, 95% CI 257.813–618.187; *p* = 0.013). BER alterations (vs. BER-proficient patients, 231.000 days, 95% CI 118.041–343.959 vs. 397.000 days, 95% CI 163.308–630.692; *p* = 0.033) indicated significantly poor PFS (**Figures 4A–C**). BER mutation also showed pooper PFS in the LADC cohort (vs. BER-proficient patients, *p* = 0.026; **Figure 4D**). For LUSC, groups with NER aberrations showed shorter PFS (*p* < 0.001) and OS (*p* = 0.02) than NER-proficient patients (**Figures 4E, F****)**. For SCLC, we observed comparably shorter PFS in patients with alterations of HR pathways (vs. HR-proficient group, 197.000 days, 95% CI 164.656–229.344 vs. 411.000 days, 95% CI 286.087–535.913; *p* = 0.04; **Figure 4G**). Although significant survival differences were observed in MMR-deficient status (*p* = 0.028) for PFS and cell cycle checkpoint-deficient status (*p* < 0.001) for OS in SCLC, and NHEJ-deficient status for PFS in LUSC (*p* < 0.001), they failed to reveal the actual situation for limited mutated samples (**Tables S7, S8**). No significant survival differences were observed in other pathways (**Tables S7, S8**). At the same time, in the whole lung cancer patients, by using univariate cox analysis, somatic alterations in single genes, including *MLH3* (HR = 3.311; 95% CI, 1.022–10.727; *p* = 0.046), *ERCC2* (HR = 15.183; 95% CI, 1.828–126.123; *p* = 0.012), *ERCC4* (HR = 15.183; 95% CI, 1.828–126.123; *p* = 0.012), *PARP2* (HR = 15.183; 95% CI, 1.828–126.123; *p* = 0.012), *BAP1* (HR = 5.234; 95% CI, 1.235–22.173; *p* = 0.025), *RAD51B* (HR = 15.183; 95% CI, 1.828–126.123; *p* = 0.012), *FANCB* (HR = 5.125; 95% CI, 1.197–21.951; *p* = 0.028), and *FANCI* (HR = 7.450; 95% CI, 1.711–32.448; *p* = 0.007; **Figure 4H**) showed statistically shorter OS after current therapy. The impacts of DDR-related single genes on patients’ OS were also identified in NSCLC and SCLC (**Figures 4I, J****)**.

### Predictive Nomogram Model Based on DDR Alterations

**Figure 5:**
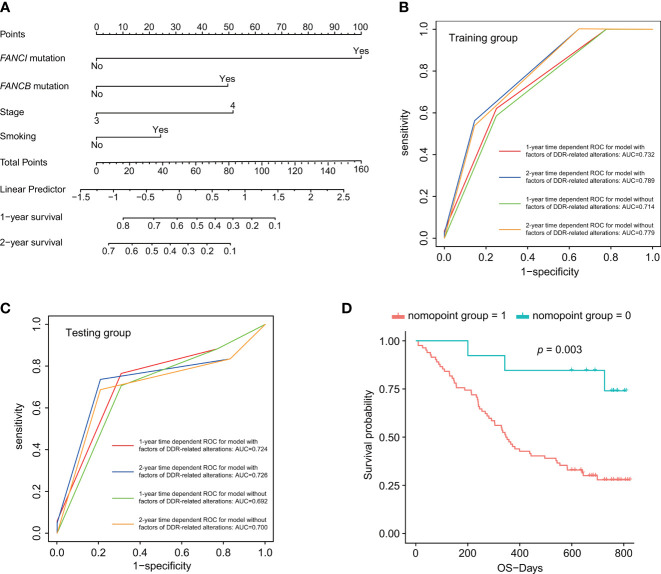
DDR-based risk score construction and predictive nomogram model. **(A)** Predictive nomogram model incorporating both clinical factors and DDR-related alterations. **(B, C)** One- and two-year time-dependent ROC curves for prediction model with or without factors of DDR-related alterations. **(D)** Survival analysis for the nomogram model. *p* < 0.05 was defined as statistically significant.

Considering the great significance of DDR pathways and single genes in predicting the prognosis of advanced lung cancer patients, we then constructed a DDR-based predictive model to predict patients’ prognosis. Apart from the univariate analysis of DDR-related alteration (**Figure 4H**), we also conducted univariate analysis based on basic clinical parameters, TMB, and MATH score (ITH), which revealed that the clinical characteristics including clinical stage (*p* < 0.001), smoking status (*p* = 0.017) were significant factors for patients’ prognosis. No significant results were obtained in different groups divided by age (*p* = 0.257), gender (*p* = 0.122), histological subtype (*p* = 0.532), TMB (p=0.337), and ITH (*p* = 0.695). Factors with significant difference were included for multivariate Cox regression analysis (**Table S9**). The Cox regression selected three independently predictive factors. Based on the result from multivariate Cox regression, a prediction model was established using the nomogram algorithm in the training dataset, which included both clinical factors and DDR-related alterations (**Figure 5A**). Smoking status was also included for model construction considering its significance to patients' prognosis. This combined prediction model showed excellent performance, with area under the curve (AUC) values of 0.732 and 0.789 for 1- and 2-year receiver operating characteristic (ROC) curves in the training group, and 0.724 and 0.726 for 1- and 2-year ROC curves in the testing group (**Figures 5B, C****)**. In addition, we also evaluated its clinical value and found that patients with high total points showed worse clinical outcomes than those with low total points (third quartile of OS, high risk vs. low risk, 188.000 days vs. 726.000 days; *p* = 0.003; **Figure 5D**). We have modified Figure 5, which was uploaded in the proof website.

### External Verification of the Mutational Characteristics of DDR and Its Clinical Values in Lung Cancer Without Driver Gene Mutations

We verified the correlations of TMB and ITH with DDR gene alterations in patients with no *EGFR* or *ALK* alterations from cBioportal database at first. Totally, consistent with our finding, higher TMB was found in DDR-deficient groups, and HR alterations were related to higher TMB in LADC, LUSC, and SCLC (**Figures S3A–C**). ITH showed significant associations with DDR altered status in LUSC and SCLC but not in LADC (**Figures S3D–F****)**. After analyzing data from public databases, high-TMB also suggested no clinical benefit in OS in LUSC (*p* = 1) and SCLC (*p* = 0.84), while for LADC, the TMB-high group demonstrated poorer OS (*p* = 0.002; **Figures S3G–I**). For ITH, only SCLC with high MATH scores showed longer survival (*p* = 0.014; **Figures S3J–L**). Then, we utilized univariate and multivariate analyses considering clinical factors and DDR-related pathway and single gene alterations and constructed a nomogram model as well in public lung cancer cohorts. Interestingly, in both LADC and SCLC public cohorts, the predictive model based on DDR gene alterations showed good performance (**Figure S4**), which were also better performed than the model without factors of DDR gene alterations.

### Immune-Related Characteristics of Lung Cancer Patients Without *EGFR/ALK* Mutations Between DDR-Deficient and DDR-Proficient Groups

**Figure 6:**
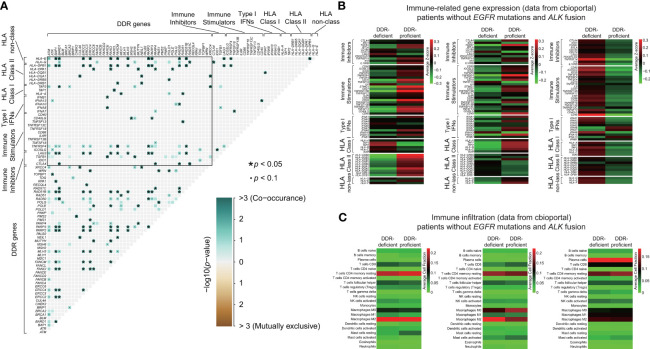
Immune-related mechanisms of lung cancer patients without *EGFR/ALK* mutations between DDR-deficient and DDR-proficient groups. **(A)** Somatic interactions of DDR genes and immunomodulatory genes. DDR genes were extensively comutated with immune genes and mutations in immune-related genes linked to specific DDR gene alterations significantly. **(B)** The expression characteristics of immune-related genes between DDR-deficient and DDR-proficient samples without *EGFR* or *ALK* mutations from cBioportal database. Samples were obtained from LADC-TCGA (n = 64), LUSC-TCGA (n = 72), and SCLC_ucologene_2015 (n = 79). **(C)** The immune microenvironment characteristics of lung cancer patients without *EGFR* mutations and *ALK* fusion from cBioportal database. Samples were obtained from LADC-TCGA (n = 64), LUSC-TCGA (n = 72), and SCLC_ucologene_2015 (n = 79). We used the R package “maftools” for the detection of co-occurring or mutually exclusive sets of genes and two-tailed Student’s test to compare immune-related gene expression between DDR-deficient and DDR-proficient subtypes *p < 0.05. •*p* < 0.1.

To elucidate underlying immune mechanisms, we first compared somatic interactions of DDR genes and immunomodulatory genes. **Figure 6A** demonstrated the extensive comutations between DDR and immune genes. Mutations in immune-related genes linked to specific DDR gene alterations significantly. Then, we analyzed the potential immune evasion caused by class I HLA genes (39). DDR gene alterations were observed comutated with various genes of HLA class I pathway including *TAP1* and *TAP2* when compared with DDR-proficient samples, although no significant difference of mutation frequency was found between the two groups (**Table S10**). We also analyzed type I interferon (IFN) genes that attach great importance to optimal immunosurveilance and antitumor efficacy. *IFNA8* and *IFNA10* mutations were observed in DDR-deficient cohort, while no relevant genes were observed in DDR-proficient cohort. However, type I IFN gene mutations were rare in our DDR-deficient cohort, with only 1 of 67 samples containing alterations in *IFNA7*, *IFNA8*, *IFNA10*, *IFNA13*, and *IFNB1* (**Table S10**). Besides, we found no difference in the mutation frequency of immune-stimulated or inhibited genes between the DDR-deficient and DDR-proficient samples (*p* > 0.05; **Table S10**). Then, we compared the expression characteristics of immune-related genes between DDR-deficient and DDR-proficient samples without *EGFR* or *ALK* mutations from the TCGA database (**Figure 6B**). **Table S11** summarized genes with *p* < 0.05. In LADC, DDR-deficient types had relatively lower expression of immune-stimulated genes (such as *STING1*, *CD28*, *HLA-DRB6*) compared with DDR-proficient types. Compared with DDR proficient-type, *TNFRSF4* (*p* = 0.017) and *TGFB1* expressions (*p* = 0.033) were lower in DDR-deficient LUSC; *CD40* (*p* = 0.012) and *TNFRSF14* expressions (*p* = 0.022) were relatively higher in DDR-deficient SCLC. Furthermore, we examined the immune microenvironment characteristics of lung cancer patients without *EGFR* mutations and *ALK* fusion (**Figure 6C**). The heatmaps suggested that immune infiltration varies with histological subtypes. Among differential immune cells (**Table S12**), DDR deficiency slightly decreased the infiltration of immune cells including resting mast cells (*p* = 0.003), memory B cells (*p* = 0.025), resting dendritic cells (*p* = 0.035) in LADC, and macrophage M0 (*p* = 0.049) in LUSC with a relatively lower cell-fraction than DDR-proficient type; activated mast cells in LADC (*p* = 0.044) and macrophage M2 in LUSC (*p* = 0.004) were slightly higher.

## Discussion

This current study mainly analyzed the mutational profiles and prognostic values of DDR pathways in the Chinese population lacking *EGFR* or *ALK* driver gene alterations. A substantial number of patients without targetable oncogenic drivers often receive chemoimmunotherapy drugs as first-line treatment. Unfortunately, few effective biomarkers are available for initial chemotherapy regimens. Acquired and inherited defects of DDR pathways are crucial mechanisms in genesis of malignancies (40). Some previous studies indicated that DDR-altered neoplasms produced favorable outcomes to platinum-based compound (17, 19). Therefore, it is important to investigate DDR-related mutational characteristics and their predictive value. We conjectured that certain DDR gene alterations might be predictive factors in lung cancer patients without oncogenic drivers. In our study, we found the extensive mutation frequency of DDR genes among these patients in which *POLQ*, *BRCA2*, *ATM*, *ATR*, *PARP4*, *POLD1* alterations were most commonly observed. MMR alterations were relatively more common in LADC (19.0%) but less frequent in SCLC (2.8%), and FA alterations were observed more frequently in LUSC (24.3%) while only 9.5% in the LADC subtype.

With the great success of immunotherapy, there remains a resurge of interest in DDR pathways with evidence showing that DDR gene alterations are positively correlated with high TMB, as a favorable biomarker for predicting response to immune-checkpoint inhibitors (10, 19, 41). Moreover, DDR mutation itself has also been identified to have putative predictive value in immunotherapy (42, 43). Our study found that deleterious DDR gene alterations were closely associated with higher TMB in comparison with proficient types, but mutations in different types of DDR pathways in diverse histopathological subtypes did not exhibit high mutational load in the same manner. Recently, higher TMB was reported frequently in tumors with altered double-strand break pathways of DDR and MMR deficiencies (41, 44, 45). It was consistent with our findings that HR alterations presented a strong relationship with high TMB in all histopathological subtypes (**Figure 2**). MMR pathway alterations were also observed associated with higher TMB in both LADC and LUSC subtypes (**Figure 2**). The mechanisms MMR deficiencies result in high TMB remain unclear, which might relate to microsatellite instability, which is important in mutation number increase *via* repeated sequences and cancer immunity alteration. Smoking was considered contributing to high TMB (46). Given the exclusion of relevant *EGFR/ALK* driver gene alterations, as many patients with oncogene target mutations were non-smokers, further study on the relationships between DDR gene alterations or smoking and TMB was conducted. We found that DDR gene alterations had a greater impact on TMB than smoking. When comparing impacts of DDR gene alterations on ITH, significant difference was only observed in LUSC patients.

To study the clinical impacts of TMB and ITH, we utilized the median value as cutoff among different lung cancer groups to detect the predictive values of TMB and MATH score among these patients. However, no survival difference was found between high and low TMB/MATH score in the whole lung cancer patients without *EGFR* or *ALK* mutations receiving first-line chemotherapy. Similar results were also identified in the SCLC population treated with platinum-based regimens alone or combined with atezolizumab, respectively, that failed to indicate the predictive prognosis value of high TMB (25, 47). These results suggested that the clinical predictive value of TMB in the sensitivity of chemotherapy and immunotherapy remains further verifications.

In contrast to our hypothesis, we identified no significant correlations among DDR gene alterations with regards to ORR and DCR (**Table S6**) and no correlation between DDR alteration status and prognosis after chemotherapy in lung cancer patients harboring no *EGFR/ALK* alterations. Hypothetically, DNA damaging chemotherapy agents contribute to DNA bending and unwinding as DNA adducts, causing apoptosis, and tumor cells with deficient DDR pathways fail to repair replication stress and show more sensitive resistance to chemotherapy (48). One explanation for this is that the co-occurrence of other genomic or epigenomic alterations with DDR mutations may dilute the influence of initial chemotherapy in lung cancer patients with no *EGFR* or *ALK* mutations. In our study, we found widespread comutations between DDR genes and significantly mutated genes. Alterations of some significantly mutated genes such as *KRAS* are well-known to be important in driving malignancy transformation and associated with worse survival (49). *KRAS* alteration was observed co-occurring with alteration of DDR gene *MDC1* in our study. We also studied the co-occurring of alterations of several important functional pathways including RTK/Ras/PI3K/AKT signaling pathway, RB pathway, and TP53 pathway (50) with DDR aberrations (**Figure S5**) and found the co-occurring of mutation of RTK/Ras/PI3K/AKT signaling pathway with cell cycle checkpoint pathway alteration (*p* < 0.05). In addition, we investigated potential immune evasion mechanisms and found that DDR-altered tumors contained enhanced frequencies of alterations in various genes of HLA class I pathway including *TAP1* and *TAP2* when compared with DDR-proficient samples.

Among DDR mechanisms, it is possible that specific DDR mechanisms could attach greater importance to the determination of the prognosis of patients after chemotherapy. Interestingly, in the analysis of specific DDR pathways in specific histological subtypes, we found that alterations of MMR, HR, and cell cycle checkpoint genes are more associated with patients’ prognosis after chemotherapy in SCLC than other DDR-related mechanisms. NER and NHEJ genes related to patients’ prognosis after chemotherapy in LUSC more, and in LADC, the relationship of BER genes and clinical outcomes was closer when compared with other DDR mechanisms. Some other publications also revealed the close relationship between MMR genes or NER expression status and platinum sensitivity (20, 48, 51). Specifically, in our data, patients with DDR gene alterations tend to have poor prognosis compared to those with intact DDR among this population. Previous research studied the effect of protein expression of DDR pathways and found that patients with low expression level were correlated with worse survival compared to those with high DDR protein expression (52). Thus, DDR-proficient type and high expression of specific pathways may benefit patients receiving chemotherapy more.

In our study, we also investigated the survival effects of single DDR genes. Among DDR genes identified, *MLH3* mutation was found associated with worse OS compared with wild-type in whole lung cancer patients. The same results were also found in genes including *FANCI*, *BAP1*, *ERCC2*, *ERCC4*, *PARP2*, *RAD51B*, and *FANCB*. However, the size of patients with these specific gene mutations was limited (n = 1), which made the results not convincing. Meanwhile, potential mechanisms of them in chemotherapy sensitivity in lung cancer were little studied, which require further exploration. We also studied the relationship between DDR mutation status and immune-related gene expression. The difference between DDR-deficient and DDR-proficient type groups was not so large, and differential genes of immune stimulatory showed inhibited expression in DDR-deficient type than DDR-proficient type in LADC and LUSC. Conversely, in SCLC, expression of both *CD40* of immune stimulatory genes and *TNFRSF14* of immune inhibitory genes was higher in DDR-deficient type than DDR proficient-type. Immune infiltration between DDR-deficient and DDR-proficient types showed very subtle differences.

Specifically, in our study, we found the great importance of alterations of DDR pathways or single genes on patients’ prognosis. Thus, we incorporated DDR-related alterations and clinical factors for the construction of a predictive nomogram model. This prediction model exhibited excellent performance for predicting patients’ survival in both the training and testing groups. Meanwhile, it could also differentiate patients with low or high risks well based on survival analysis. The addition of DDR-related alterations could enhance the performance of the model to better predict patients’ prognosis, which was also verified in public datasets. In addition, we also verified the relationships of DDR gene alterations with TMB, ITH, and survival and found many similar results with our own dataset.

There are several limitations of our study. The median 754-day follow-up time may not be enough to investigate the long-term survival rate. Ideally, all patients should be followed up for more than 5 years. In our study, we found that worse PFS was frequent in specific DDR-altered lung cancer patients without *EGFR* or *ALK* mutations. Although several explanations were investigated, the underlying mechanisms require further exploration. Furthermore, deleterious DDR gene alterations might not be sufficiently extensive to study prognosis in specific histological subtypes such as limited samples of MMR- or cell cycle checkpoint-related mutations in SCLC. Therefore, we utilized external cBioportal database for verification to enhance the credibility of our results. The frequency of alterations of several genes was extremely low (n = 1), which made the mutation occurrence analysis not convincing. Larger studies involving more patients should be conducted for verification.

## Conclusion

This current study mainly focused on mutational profiles and prognostic values of DDR pathways in the Chinese population lacking *EGFR* or *ALK* driver gene alterations. DDR gene alterations are positively associated with high TMB among these patients. Specific DDR gene alterations tend to associate with worse progression-free survival to initial therapy. Meanwhile, the immune heterogeneity of different molecules and infiltrating cells were also revealed. These results will enable discoveries of promising prognostic biomarkers and potential therapeutic targets for lung cancer patients without oncogenic drivers.

## Data Availability Statement

The datasets presented in this study can be found in the article/**Supplementary Material**.

## Ethics Statement

The studies involving human participants were reviewed and approved by the ethics committee of the Shanghai Pulmonary Hospital. The patients/participants provided their written informed consent to participate in this study.

## Author Contributions

JD, MJ, HL, and YH conceived the idea of this paper. JD, MJ, KH, HW, PC, HG, WZ, HL, and YH participated in the acquisition and treatment of data. JD, MJ, KH, HW, PC, and HG, and WZ implemented the analysis. JD, MJ, KH, HW, PC, HG, WZ, HL, YH, and CZ contributed to the writing of the manuscript. All authors contributed to the article and approved the submitted version.

## Funding

This study was supported in part by National Key R&D Program of China (2018YFC0910500), the Neil Shen’s SJTU Medical Research Fund, National Natural Science Foundation of China (81802255), Young Talents in Shanghai (2019 QNBJ), “Dream Tutor” Outstanding Young Talents Program (fkyq1901), Clinical Research Project of Shanghai Pulmonary Hospital (fk18005), Key Discipline in 2019 (oncology), Project of Shanghai Municipal Science and Technology Commission (Project of Municipal Science and Technology Commission), Scientific Research Project of Shanghai Pulmonary Hospital (fkcx1903), Shanghai Municipal Commission of Health and Family Planning (2017YQ050), Innovation Training Project of SITP of Tongji University, and key projects of leading talent (19411950300).

## Conflict of Interest

The authors declare that the research was conducted in the absence of any commercial or financial relationships that could be construed as a potential conflict of interest.

## Publisher’s Note

All claims expressed in this article are solely those of the authors and do not necessarily represent those of their affiliated organizations, or those of the publisher, the editors and the reviewers. Any product that may be evaluated in this article, or claim that may be made by its manufacturer, is not guaranteed or endorsed by the publisher.
