# Supplementary material for: DNA Damage Response and Repair Gene Alterations Increase Tumor Mutational Burden and Promote Poor Prognosis of Advanced Lung Cancer
Source: Front Oncol. 2021 Sep 15;11:708294. doi: 10.3389/fonc.2021.708294 (PMC8479169; doi:10.3389/fonc.2021.708294)
Supplement: Supplementary file 1 [file DataSheet_1.docx]

Supplementary Material

# Supplementary Figures and Tables

## Supplementary Figures


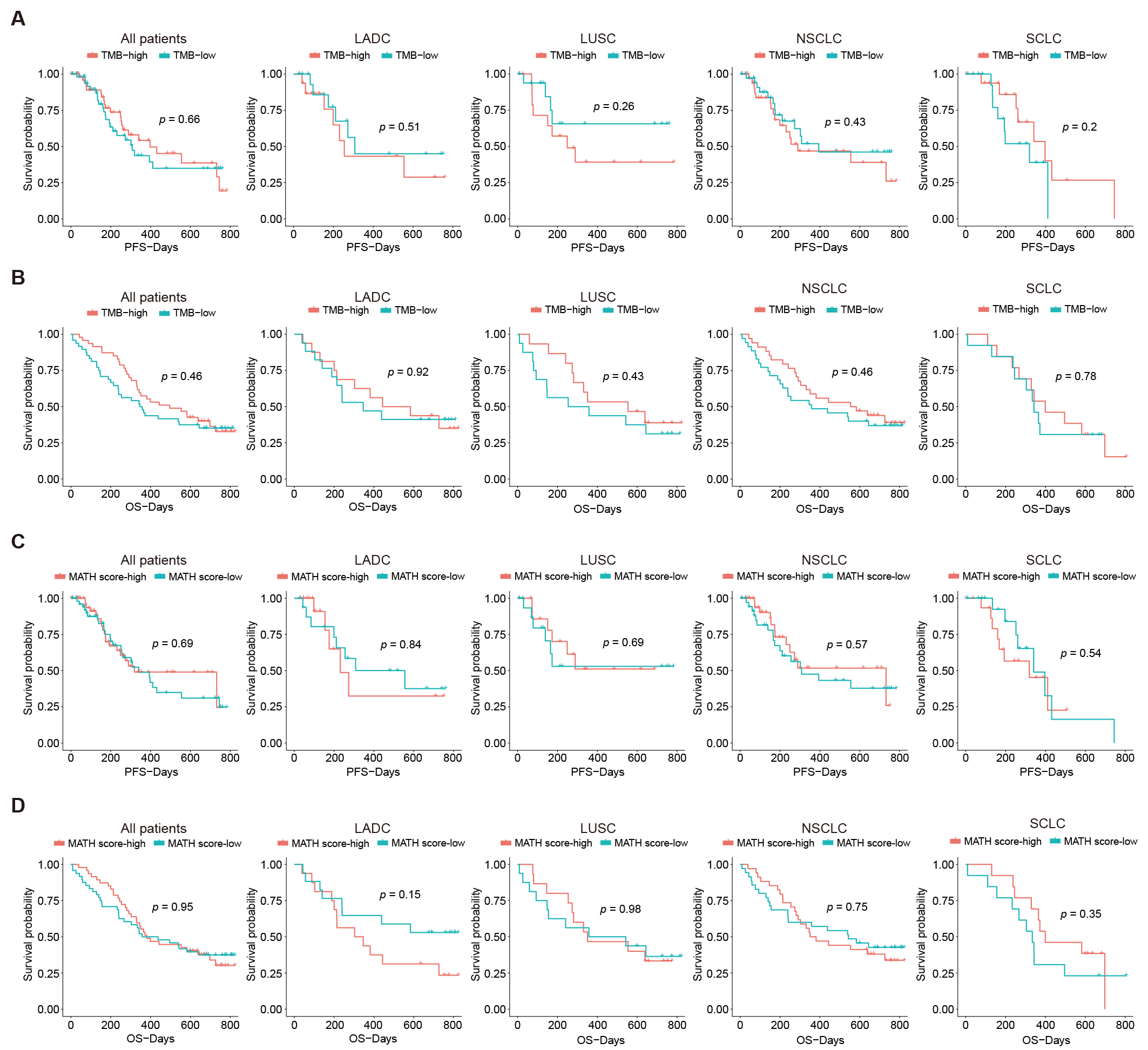


**Figure S1. Effect of patient TMB level and MATH score on PFS and OS in lung cancer patients without *EGFR/ALK* alterations.** **(A).** Survival analyses of TMB level on PFS in all patients, in LADC, LUSC, NSCLC, and SCLC cohorts respectively. **(B).** Survival analyses of TMB level on OS in all patients, in LADC, LUSC, NSCLC, and SCLC cohorts respectively. **(C).** Survival analyses of MATH score on PFS in all patients, in LADC, LUSC, NSCLC, and SCLC cohorts respectively. **(D).** Survival analyses of MATH score on OS in all patients, in LADC, LUSC, NSCLC, and SCLC cohorts respectively. Kaplan-Meier survival analysis was used to evaluate the impacts of TMB level and MATH score on PFS and OS and log-rank test was used for comparison.

**
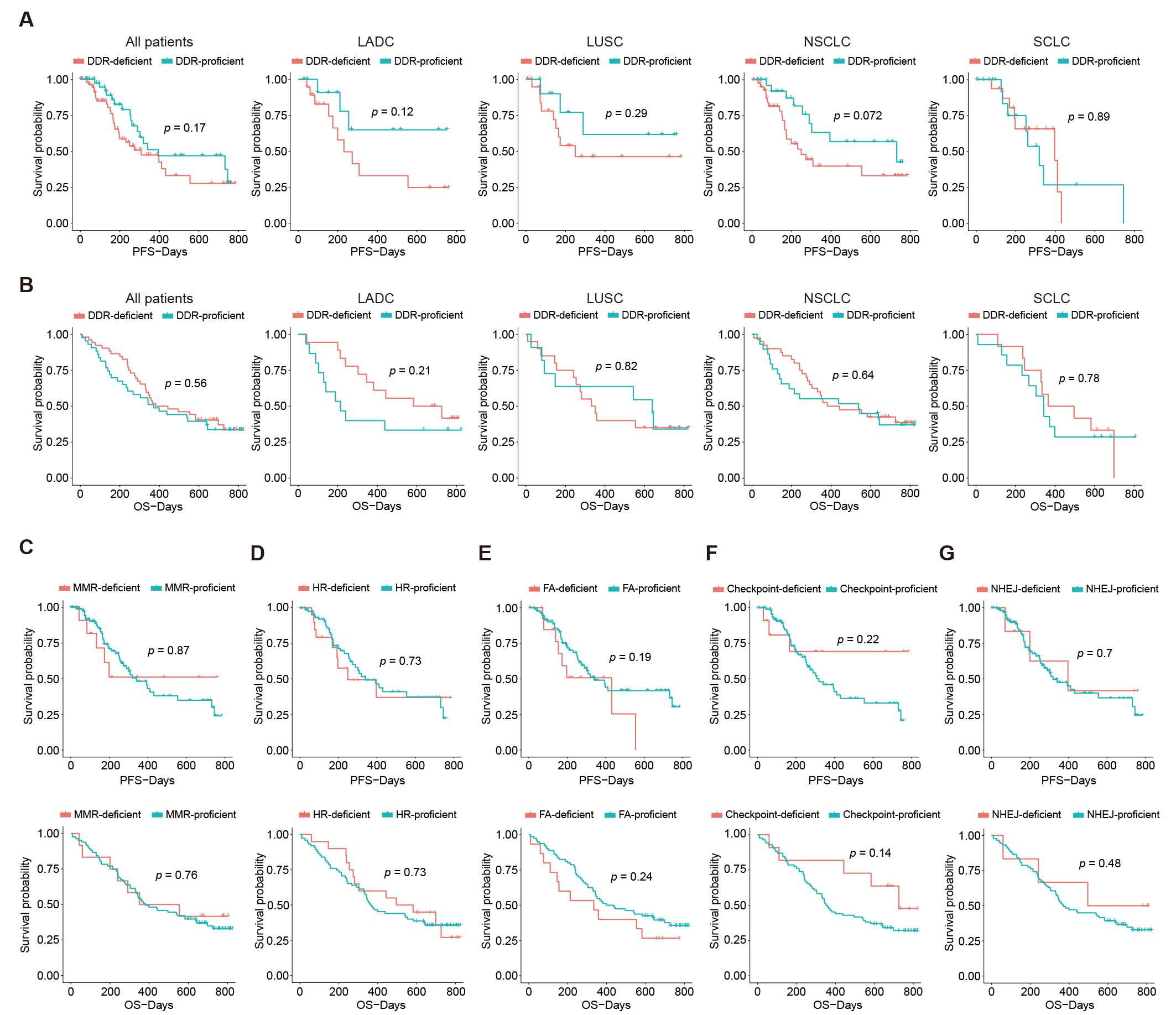
**

**Figure S2. Effects of DDR gene alterations on PFS and OS in patients without *EGFR/ALK* mutations receiving first-line chemotherapy. (A).** Survival analyses of DDR alterations on PFS in all patients, as well as in LADC, LUSC, NSCLC, and SCLC cohorts respectively. **(B).** Survival analyses of DDR alterations on OS in all patients, as well as in LADC, LUSC, NSCLC, and SCLC cohorts respectively. No clinical association was found when analyzing PFS and OS in distinct cohorts between DDR-deficient with DDR-proficient patients. **(C-G).** Survival analyses of DDR alterations on PFS and OS in MMR, HR, FA, Checkpoint, and NHEJ pathways in all patients. Kaplan-Meier survival analysis was used to evaluate the impacts of DDR gene alterations on PFS and OS and log-rank test was used for comparison.


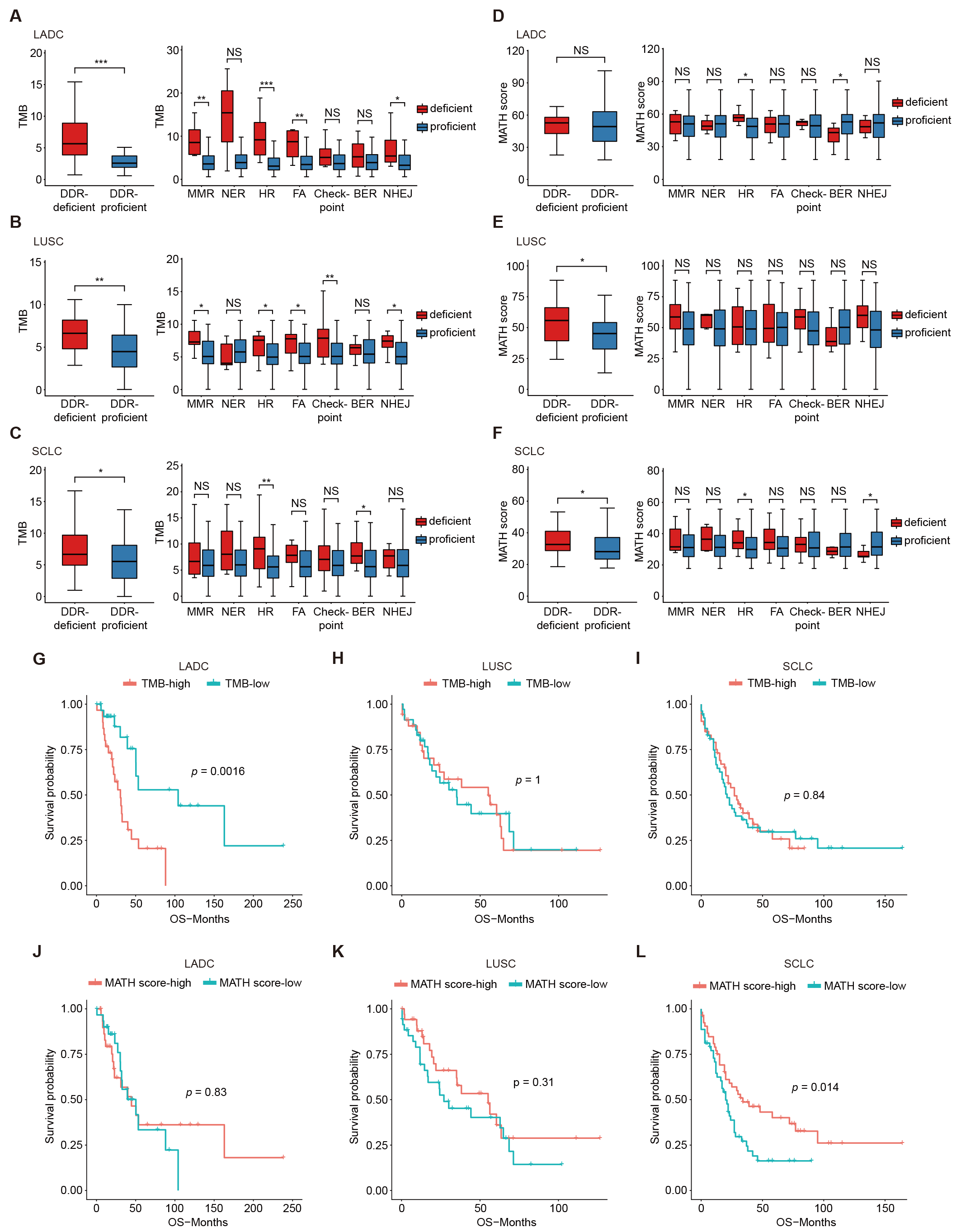


**Figure S3. External verification of DDR and its association with TMB, ITH, and survival. (A)**. Correlations of TMB with DDR alterations in LADC patients with no *EGFR* or *ALK* alterations from cBioportal database (LADC-TCGA, n=64). **(B)**. Correlations of TMB with DDR alterations in LUSC patients with no *EGFR* or *ALK* alterations from cBioportal database (LUSC-TCGA, n=72). **(C)**. Correlations of TMB with DDR alterations in SCLC patients with no *EGFR* or *ALK* alterations from cBioportal database (sclc_ucologne_2015, n=116). **(D)**. Correlations of MATH score with DDR alterations in LADC patients with no *EGFR* or *ALK* alterations from cBioportal database (LADC-TCGA, n=64). **(E).** Correlations of MATH score with DDR alterations in LUSC patients with no *EGFR* or *ALK* alterations from cBioportal database (LUSC-TCGA, n=72). **(F).** Correlations of MATH score with DDR alterations in SCLC patients with no *EGFR* or *ALK* alterations from cBioportal database (sclc_ucologne_2015, n=116). **(G-I).** Impacts of TMB on OS in LADC, LUSC, and SCLC patients without EGFR/ALK alterations. **(J-L).** Impacts of MATH score on OS in LADC, LUSC, and SCLC patients without EGFR/ALK alterations. Wilcoxon rank-sum test was utilized to study the relationship of DDR gene alterations with TMB and MATH score. Kaplan-Meier survival analysis was used to evaluate the impacts of levels of TMB and MATH score on PFS and OS and log-rank test was used for comparison.


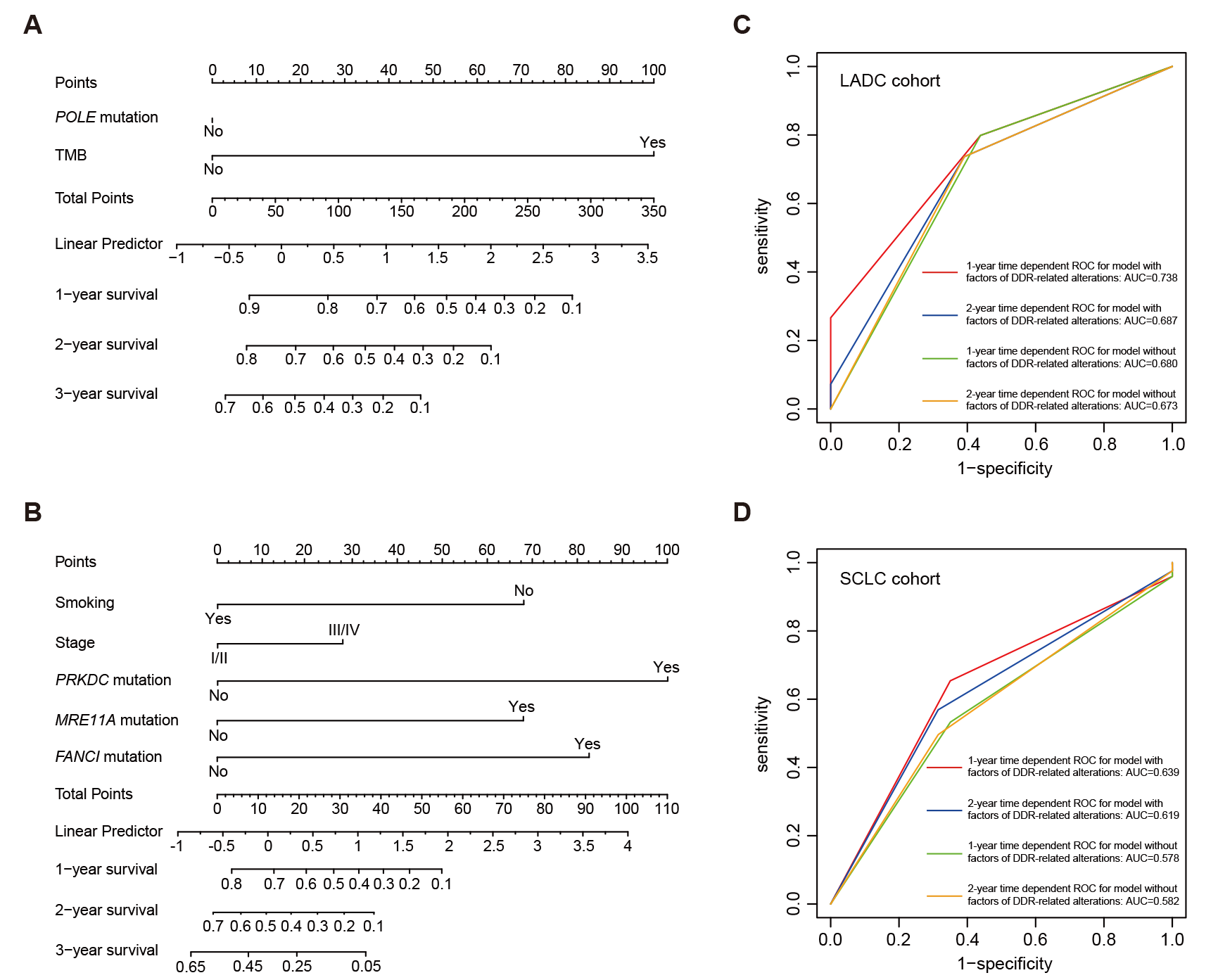


**Figure S4. The predictive model based on DDR alterations using public datasets. (A-B)**. Nomogram model in LADC public cohort and SCLC public cohort respectively. **(C-D)**. Time-dependent ROC curves in the LADC public cohort and SCLC public cohort respectively.


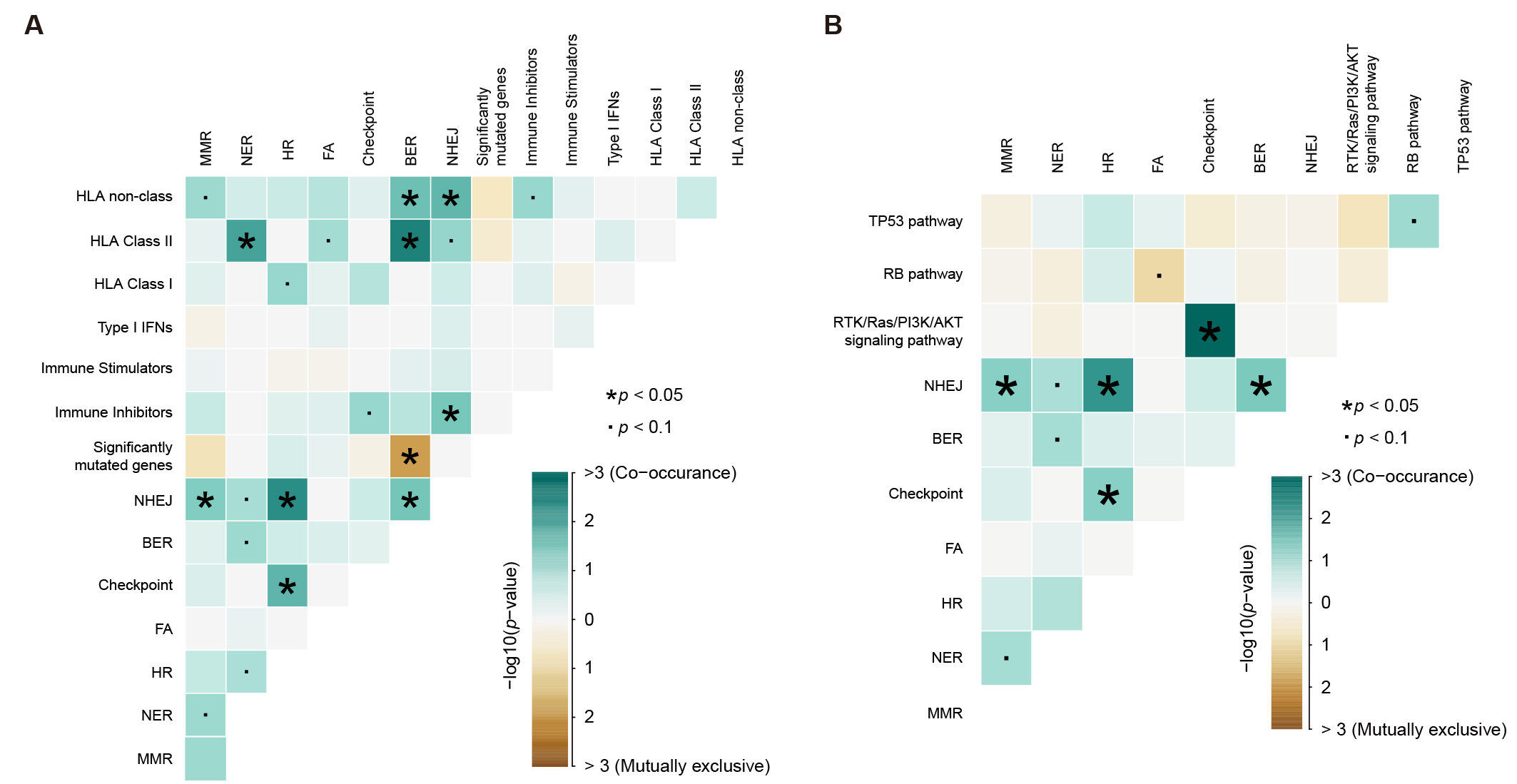


**Figure S5.** **The co-occurring of alterations of some important signaling pathway with DDR pathway alterations.** A. The co-occurring of alterations of HLA, type I IFN signaling pathway and immune related gene sets with DDR gene alterations. B. The co-occurring of alterations of several import functional pathways including RTK/Ras/PI3K/AKT signaling pathway, RB pathway, and TP53 pathway with DDR aberrations.

## Supplementary Tables

**Table S1. The clinical characteristics of patients**

| **Clinical Characteristics** | All (n=122) |
| --- | --- |
| **Age** |  |
| <65 | 51 |
| ≥65 | 71 |
| **Gender** |  |
| Male | 106 |
| Female | 16 |
| **Smoking status** |  |
| Smoker | 85 |
| Non-smoker | 37 |
| **Response** |  |
| PR | 27 |
| SD | 57 |
| PD | 13 |
| NA | 25 |

Note: PD, progressive disease; PR, partial response; SD, stable disease; LADC, lung adenocarcinoma; LUSC, lung squamous cell carcinoma; SCLC, small cell lung cancer.

| **Table S2. DDR-related genes and functional pathways enrolled** | | | | | | |
| --- | --- | --- | --- | --- | --- | --- |
| **MMR** | **NER** | **HR** | **FA** | **Cell-cycle checkpoint** | **BER** | **NHEJ** |
| MLH1 | CUL4A | BAP1 | BLM | ATM | APEX1 | LIG4 |
| MLH3 | ERCC1 | BARD1 | BRIP1 | ATR | MUTYH | NHEJ1 |
| MSH2 | ERCC2 | BLM | FANCA | CHEK1 | NEIL1 | PARP3 |
| MSH6 | ERCC3 | BRCA1 | FANCB | CHEK2 | PARP1 | POLL |
| PMS1 | ERCC4 | BRCA2 | FANCC | MDC1 | PARP2 | POLM |
| PMS2 | ERCC5 | BRIP1 | FANCD2 | TOPBP1 | PARP3 | PRKDC |
| RPA1 | ERCC6 | MRE11A | FANCE | WEE1 | PARP4 | RAD50 |
|  | RPA1 | NBN | FANCF |  | PNKP | XRCC4 |
|  |  | PALB2 | FANCG |  | POLB | XRCC5 |
|  |  | POLQ | FANCI |  | POLD1 | XRCC6 |
|  |  | RAD50 | FANCL |  | POLE |  |
|  |  | RAD51 | FANCM |  | TDG |  |
|  |  | RAD51B | USP1 |  | XRCC1 |  |
|  |  | RAD51C |  |  |  |  |
|  |  | RAD51D |  |  |  |  |
|  |  | RAD52 |  |  |  |  |
|  |  | RAD54B |  |  |  |  |
|  |  | RAD54L |  |  |  |  |
|  |  | RECQL4 |  |  |  |  |
|  |  | WRN |  |  |  |  |
|  |  | XRCC3 |  |  |  |  |

Note: A total of 74 DDR genes were assembled as being associated with DDR, grouped into different functional pathways from published resources^15,19,23-27^.

**Table S3. Deleterious alterations identified**

| **Patient** | **Gene** | **AA change** | **Deleterious_by_loss_of_function** | **Deleterious_by_in_silico_analysis** | **Deleterious_by_manual_review** | **Type_of_Alterations** |
| --- | --- | --- | --- | --- | --- | --- |
| Patient5 | ATR | H506Q |  | 1 | 0 | Missense_Mutation |
| Patient5 | BRCA2 | D3095Y |  | 1 | 0 | Missense_Mutation |
| Patient10 | RAD50 | Q975Lfs*6 | 1 |  |  | Frame_Shift_Ins |
| Patient12 | MSH6 | D499Mfs*6 | 1 |  |  | Frame_Shift_Del |
| Patient12 | MSH6 | K502Qfs*8 | 1 |  |  | Frame_Shift_Ins |
| Patient12 | PARP3 | R110P |  | 1 | 0 | Missense_Mutation |
| Patient13 | ERCC3 | C512Y |  | 1 | 0 | Missense_Mutation |
| Patient14 | RAD51 | D223H |  | 1 | 0 | Missense_Mutation |
| Patient15 | PNKP | W331X | 1 |  |  | Nonsense_Mutation |
| Patient16 | BAP1 | V704A |  | 1 | 0 | Missense_Mutation |
| Patient16 | RPA1 | A9V |  | 1 | 0 | Missense_Mutation |
| Patient17 | FANCD2 | A799S |  | 1 | 0 | Missense_Mutation |
| Patient18 | PARP4 | E298Q |  | 1 | 0 | Missense_Mutation |
| Patient18 | FANCA | E440D |  | 1 | 0 | Missense_Mutation |
| Patient21 | MSH6 | D499Mfs*6 | 1 |  |  | Frame_Shift_Del |
| Patient21 | POLD1 | T412Pfs*66 | 1 |  |  | Frame_Shift_Del |
| Patient24 | MSH6 | D499Mfs*6 | 1 |  |  | Frame_Shift_Del |
| Patient24 | XRCC4 | L113Ffs*3 | 1 |  |  | Frame_Shift_Ins |
| Patient24 | XRCC4 | E114Gfs*2 | 1 |  |  | Frame_Shift_Del |
| Patient24 | ATM | E2468Dfs*7 | 1 |  |  | Frame_Shift_Del |
| Patient24 | ATM | I2471Nfs*12 | 1 |  |  | Frame_Shift_Ins |
| Patient24 | BRCA2 | S1632Rfs*4 | 1 |  |  | Frame_Shift_Del |
| Patient24 | BRCA2 | F1634Lfs*5 | 1 |  |  | Frame_Shift_Ins |
| Patient27 | ATM | A920Qfs*9 | 1 |  |  | Frame_Shift_Del |
| Patient27 | BRCA1 | C1223F |  | 1 | 0 | Missense_Mutation |
| Patient28 | PMS1 | L414V |  | 1 | 0 | Missense_Mutation |
| Patient30 | PNKP | A428T |  | 0 | 1 | Missense_Mutation |
| Patient32 | FANCA | E918X | 1 |  |  | Nonsense_Mutation |
| Patient33 | FANCD2 | S35C |  | 1 | 1 | Missense_Mutation |
| Patient35 | MDC1 | G1005D |  | 1 | 0 | Missense_Mutation |
| Patient35 | RAD51 | Q6X | 1 |  |  | Nonsense_Mutation |
| Patient35 | POLD1 | T412Pfs*66 | 1 |  |  | Frame_Shift_Del |
| Patient37 | TDG | K58T |  | 1 | 1 | Missense_Mutation |
| Patient39 | MUTYH | R217Efs*48 | 1 |  |  | Frame_Shift_Del |
| Patient39 | ERCC6 | Y1155_C1493del | 1 |  |  | Nonsense_Mutation |
| Patient39 | POLE | A944T |  | 1 | 0 | Missense_Mutation |
| Patient39 | PARP4 | F324V |  | 1 | 0 | Missense_Mutation |
| Patient39 | FANCI | V859G |  | 1 | 0 | Missense_Mutation |
| Patient40 | FANCE | A40P |  | 1 | 0 | Missense_Mutation |
| Patient45 | ATM | P113T |  | 1 | 0 | Missense_Mutation |
| Patient45 | FANCB | Y93N |  | 1 | 0 | Missense_Mutation |
| Patient48 | BRCA2 | D224N |  | 1 | 1 | Missense_Mutation |
| Patient48 | BRIP1 | Q168Hfs*26 | 1 |  |  | Frame_Shift_Del |
| Patient49 | POLE | S1829F |  | 1 | 0 | Missense_Mutation |
| Patient51 | MLH3 | S1353T |  | 1 | 0 | Missense_Mutation |
| Patient52 | FANCA | R880X | 1 |  |  | Nonsense_Mutation |
| Patient54 | POLQ | L229M |  | 1 | 0 | Missense_Mutation |
| Patient56 | POLQ | R2241X | 1 |  |  | Nonsense_Mutation |
| Patient56 | ATM | Y1915C |  | 1 | 0 | Missense_Mutation |
| Patient58 | BARD1 | E298* | 1 |  |  | Nonsense_Mutation |
| Patient58 | PARP3 | H441P |  | 1 | 0 | Missense_Mutation |
| Patient58 | BAP1 | V704A |  | 1 | 0 | Missense_Mutation |
| Patient58 | RAD50 | Q975Lfs*6 | 1 |  |  | Frame_Shift_Ins |
| Patient58 | ATM | D2050N |  | 1 | 0 | Missense_Mutation |
| Patient58 | PARP2 | E545Ifs*5 | 1 |  |  | Frame_Shift_Del |
| Patient58 | PARP2 | I547Sfs*15 | 1 |  |  | Frame_Shift_Ins |
| Patient58 | FANCM | F285* | 1 |  |  | Nonsense_Mutation |
| Patient58 | RAD51B | C27X | 1 |  |  | Nonsense_Mutation |
| Patient58 | MLH3 | F253Lfs*5 | 1 |  |  | Frame_Shift_Del |
| Patient58 | FANCI | V1156Lfs*9 | 1 |  |  | Frame_Shift_Ins |
| Patient58 | FANCI | N1158* | 1 |  |  | Nonsense_Mutation |
| Patient58 | ERCC4 | E636Dfs*7 | 1 |  |  | Frame_Shift_Del |
| Patient58 | ERCC2 | Y584D |  | 1 | 0 | Missense_Mutation |
| Patient58 | ERCC2 | E582G |  | 1 | 0 | Missense_Mutation |
| Patient59 | FANCM | S1027L |  | 0 | 1 | Missense_Mutation |
| Patient60 | ERCC3 | R174G |  | 1 | 0 | Missense_Mutation |
| Patient60 | RECQL4 | K136N |  | 1 | 0 | Missense_Mutation |
| Patient62 | FANCM | G74R |  | 1 | 1 | Missense_Mutation |
| Patient62 | NEIL1 | T4Sfs*15 | 1 |  |  | Frame_Shift_Ins |
| Patient62 | NEIL1 | R7Sfs*91 | 1 |  |  | Frame_Shift_Del |
| Patient64 | ATR | D730H |  | 1 | 0 | Missense_Mutation |
| Patient67 | ERCC2 | R601W |  | 1 | 1 | Missense_Mutation |
| Patient68 | POLD1 | L50Q |  | 1 | 0 | Missense_Mutation |
| Patient73 | XRCC4 | K26N |  | 1 | 0 | Missense_Mutation |
| Patient73 | CUL4A | Y453C |  | 1 | 0 | Missense_Mutation |
| Patient73 | CUL4A | M674I |  | 1 | 0 | Missense_Mutation |
| Patient73 | NEIL1 | T4Sfs*15 | 1 |  |  | Frame_Shift_Ins |
| Patient74 | TOPBP1 | R1003Q |  | 1 | 0 | Missense_Mutation |
| Patient74 | ATR | C2272Y |  | 1 | 0 | Missense_Mutation |
| Patient76 | MLH3 | D318N |  | 1 | 0 | Missense_Mutation |
| Patient77 | POLQ | W1946C |  | 1 | 0 | Missense_Mutation |
| Patient77 | MDC1 | F326L |  | 1 | 0 | Missense_Mutation |
| Patient77 | POLE | D1214Y |  | 1 | 0 | Missense_Mutation |
| Patient77 | POLE | D83N |  | 1 | 0 | Missense_Mutation |
| Patient78 | BRCA2 | Q1138H |  | 1 | 0 | Missense_Mutation |
| Patient79 | FANCA | L60P |  | 1 | 0 | Missense_Mutation |
| Patient81 | FANCB | L23I |  | 1 | 0 | Missense_Mutation |
| Patient82 | PMS2 | G150S |  | 1 | 0 | Missense_Mutation |
| Patient82 | CHEK2 | A392Sfs*31 | 1 |  |  | Frame_Shift_Ins |
| Patient84 | TDG | M184Gfs*8 | 1 |  |  | Frame_Shift_Ins |
| Patient85 | ATR | H2527R |  | 1 | 0 | Missense_Mutation |
| Patient86 | FANCE | A401T |  | 1 | 0 | Missense_Mutation |
| Patient88 | MLH1 | L51P |  | 1 | 0 | Missense_Mutation |
| Patient89 | ATR | F1518Yfs*2 | 1 |  |  | Nonsense_Mutation |
| Patient89 | PNKP | A158P |  | 1 | 0 | Missense_Mutation |
| Patient90 | POLQ | R2169P |  | 1 | 0 | Missense_Mutation |
| Patient90 | BLM | L258V |  | 1 | 0 | Missense_Mutation |
| Patient91 | MDC1 | T1198N |  | 1 | 0 | Missense_Mutation |
| Patient92 | MSH6 | L60Sfs*27 | 1 |  |  | Frame_Shift_Ins |
| Patient92 | FANCE | M473I |  | 1 | 0 | Missense_Mutation |
| Patient93 | FANCL | K88X | 1 |  |  | Nonsense_Mutation |
| Patient93 | FANCL | L87V |  | 1 | 0 | Missense_Mutation |
| Patient97 | BARD1 | E251X | 1 |  |  | Nonsense_Mutation |
| Patient97 | RAD50 | K721Gfs*5 | 1 |  |  | Frame_Shift_Del |
| Patient97 | PARP4 | L1084Sfs*18 | 1 |  |  | Frame_Shift_Del |
| Patient97 | PARP4 | R1083P |  | 1 | 0 | Missense_Mutation |
| Patient97 | RAD51C | Q285H |  | 1 | 0 | Missense_Mutation |
| Patient101 | PMS1 | G474R |  | 1 | 0 | Missense_Mutation |
| Patient101 | POLQ | M136I |  | 0 | 1 | Missense_Mutation |
| Patient102 | PARP4 | P1049L |  | 1 | 1 | Missense_Mutation |
| Patient104 | POLQ | N1672K |  | 1 | 0 | Missense_Mutation |
| Patient104 | RAD50 | N90D |  | 1 | 0 | Missense_Mutation |
| Patient105 | PMS1 | W484C |  | 1 | 0 | Missense_Mutation |
| Patient105 | POLQ | S1821I |  | 1 | 0 | Missense_Mutation |
| Patient105 | WRN | S1058C |  | 1 | 0 | Missense_Mutation |
| Patient105 | ERCC4 | D834Y |  | 1 | 0 | Missense_Mutation |
| Patient105 | POLD1 | C899G |  | 1 | 0 | Missense_Mutation |
| Patient106 | TDG | Q112H |  | 1 | 0 | Missense_Mutation |
| Patient109 | MSH2 | D506H |  | 1 | 0 | Missense_Mutation |
| Patient111 | BARD1 | E209X | 1 |  |  | Nonsense_Mutation |
| Patient112 | ATM | K952Rfs*19 | 1 |  |  | Frame_Shift_Del |
| Patient113 | PALB2 | S357Y |  | 1 | 0 | Missense_Mutation |
| Patient114 | ERCC6 | S268I |  | 1 | 0 | Missense_Mutation |
| Patient117 | BRCA2 | R155I |  | 1 | 1 | Missense_Mutation |
| Patient117 | POLD1 | G395V |  | 1 | 0 | Missense_Mutation |
| Patient118 | BRCA2 | L24I |  | 1 | 0 | Missense_Mutation |
| Patient120 | TDG | A267D |  | 1 | 0 | Missense_Mutation |
| Patient120 | BRIP1 | T166Lfs*28 | 1 |  |  | Frame_Shift_Del |
| Patient121 | PARP4 | I1039T |  | 1 | 1 | Missense_Mutation |
| Patient122 | RECQL4 | D424H |  | 1 | 0 | Missense_Mutation |
| Patient122 | RECQL4 | D157Y |  | 1 | 0 | Missense_Mutation |

AA change, amino acid change.

**Table S4. Associations between TMB levels and genomic alterations of DDR pathways**

|  | **Alteration status** | | **odds ratio** | **P** |
| --- | --- | --- | --- | --- |
|  | **Negative** | **Positive** |  |  |
| **DDR** |  |  |  |  |
| Low TMB | 35 | 26 | 2.76 | **0.006** |
| High TMB | 20 | 41 |  |  |
| **MMR** |  |  |  |  |
| Low TMB | 57 | 4 | 2.794 | 0.088 |
| High TMB | 51 | 10 |  |  |
| **NER** |  |  |  |  |
| Low TMB | 59 | 2 | 3.824 | 0.166 |
| High TMB | 54 | 7 |  |  |
| **HR** |  |  |  |  |
| Low TMB | 54 | 7 | 3.229 | **0.014** |
| High TMB | 43 | 18 |  |  |
| **FA** |  |  |  |  |
| Low TMB | 54 | 7 | 1.889 | 0.212 |
| High TMB | 49 | 12 |  |  |
| **Cell-cycle checkpoint** |  |  |  |  |
| Low TMB | 58 | 3 | 4.735 | **0.013** |
| High TMB | 49 | 12 |  |  |
| **BER** |  |  |  |  |
| Low TMB | 52 | 9 | 1.721 | 0.247 |
| High TMB | 47 | 14 |  |  |
| **NHEJ** |  |  |  |  |
| Low TMB | 60 | 55 | 6.545 | 0.119 |
| High TMB | 1 | 6 |  |  |

Note: BER, base excision repair; DDR, DNA damage response and repair; FA, Fanconi anemia; HR, homologous recombination; MMR, mismatch repair; NER, nucleotide excision repair; NHEJ, non-homologous end joining; TMB, tumor mutation burden.

**Table S5. Alterations between ITH levels and genomic alterations of DDR**

|  | **Alteration status** | | **odds ratio** | **P** |
| --- | --- | --- | --- | --- |
|  | **Negative** | **Positive** |  |  |
| **DDR** |  |  |  |  |
| Low MATH score | 31 | 30 | 1.593 | 0.203 |
| High MATH score | 24 | 37 |  |  |
| **MMR** |  |  |  |  |
| Low MATH score | 53 | 8 | 0.723 | 0.57 |
| High MATH score | 55 | 6 |  |  |
| **NER** |  |  |  |  |
| Low MATH score | 57 | 4 | 1.272 | 1 |
| High MATH score | 56 | 5 |  |  |
| **HR** |  |  |  |  |
| Low MATH score | 51 | 10 | 1.663 | 0.262 |
| High MATH score | 46 | 15 |  |  |
| **FA** |  |  |  |  |
| Low MATH score | 51 | 10 | 0.883 | 0.803 |
| High MATH score | 52 | 9 |  |  |
| **Cell-cycle checkpoint** |  |  |  |  |
| Low MATH score | 51 | 10 | 0.455 | 0.168 |
| High MATH score | 56 | 5 |  |  |
| **BER** |  |  |  |  |
| Low MATH score | 50 | 11 | 1.113 | 0.817 |
| High MATH score | 49 | 12 |  |  |
| **NHEJ** |  |  |  |  |
| Low MATH score | 55 | 6 | 0.153 | 0.119 |
| High MATH score | 60 | 1 |  |  |

Note: BER, base excision repair; DDR, DNA damage response and repair; FA, Fanconi anemia; HR, homologous recombination; ITH, intratumor heterogeneity; MMR, mismatch repair; NER, nucleotide excision repair; NHEJ, non-homologous end joining.

**Table S6. Correlations among DDR alterations with regards to ORR and DCR**

|  | **DDR** |  |  |  |  | **DDR** |  |  |  |
| --- | --- | --- | --- | --- | --- | --- | --- | --- | --- |
|  | (-) n = 43 | (+) n = 54 | odds ratio | p |  | (-) n = 43 | (+) n = 54 | odds ratio | p |
| non-DCR | 5 | 8 | 0.757 | 0.647 | non-ORR | 30 | 40 | 0.808 | 0.638 |
| DCR | 38 | 46 |  |  | ORR | 13 | 14 |  |  |
|  |  |  |  |  |  |  |  |  |  |
|  | **MMR** |  |  |  |  | **MMR** |  |  |  |
|  | (-) n = 86 | (+) n = 11 | odds ratio | p |  | (-) n = 86 | (+) n = 11 | odds ratio | p |
| non-DCR | 11 | 2 | 0.66 | 0.981 | non-ORR | 61 | 9 | 0.542 | 0.688 |
| DCR | 75 | 9 |  |  | ORR | 25 | 2 |  |  |
|  |  |  |  |  |  |  |  |  |  |
|  | **NER** |  |  |  |  | **NER** |  |  |  |
|  | (-) n = 89 | (+) n = 8 | odds ratio | p |  | (-) n = 89 | (+) n = 8 | odds ratio | p |
| non-DCR | 12 | 1 | 1.091 | 0.643 | non-ORR | 63 | 7 | 0.346 | 0.549 |
| DCR | 77 | 7 |  |  | ORR | 26 | 1 |  |  |
|  |  |  |  |  |  |  |  |  |  |
|  | **HR** |  |  |  |  | **HR** |  |  |  |
|  | (-) n = 79 | (+) n = 18 | odds ratio | p |  | (-) n = 79 | (+) n = 18 | odds ratio | p |
| non-DCR | 9 | 4 | 0.45 | 0.404 | non-ORR | 57 | 13 | 0.997 | 0.995 |
| DCR | 70 | 14 |  |  | ORR | 22 | 5 |  |  |
|  |  |  |  |  |  |  |  |  |  |
|  | **FA** |  |  |  |  | **FA** |  |  |  |
|  | (-) n = 83 | (+) n = 14 | odds ratio | p |  | (-) n = 83 | (+) n = 14 | odds ratio | p |
| non-DCR | 11 | 2 | 0.917 | 0.750 | non-ORR | 60 | 10 | 1.043 | 0.798 |
| DCR | 72 | 12 |  |  | ORR | 23 | 4 |  |  |
|  |  |  |  |  |  |  |  |  |  |
|  | **Cell-cycle checkpoint** | |  |  |  | **Cell-cycle checkpoint** | |  |  |
|  | (-) n = 86 | (+) n = 11 | odds ratio | p |  | (-) n = 86 | (+) n = 11 | odds ratio | p |
| non-DCR | 11 | 2 | 0.66 | 0.981 | non-ORR | 62 | 8 | 0.969 | 0.754 |
| DCR | 75 | 9 |  |  | ORR | 24 | 3 |  |  |
|  |  |  |  |  |  |  |  |  |  |
|  | **BER** |  |  |  |  | **BER** |  |  |  |
|  | (-) n = 78 | (+) n = 19 | odds ratio | p |  | (-) n = 78 | (+) n = 19 | odds ratio | p |
| non-DCR | 10 | 3 | 0.784 | 0.972 | non-ORR | 55 | 15 | 0.638 | 0.462 |
| DCR | 68 | 16 |  |  | ORR | 23 | 4 |  |  |
|  |  |  |  |  |  |  |  |  |  |
|  | **NHEJ** |  |  |  |  | **NHEJ** |  |  |  |
|  | (-) n = 92 | (+) n = 5 | odds ratio | p |  | (-) n = 92 | (+) n = 5 | odds ratio | p |
| non-DCR | 13 | 0 | inf | 1 | non-ORR | 66 | 4 | 0.635 | 0.912 |
| DCR | 79 | 5 |  |  | ORR | 26 | 1 |  |  |

Note: BER, base excision repair; DCR, disease control rate; DDR, DNA damage response and repair; FA, Fanconi anemia; HR, homologous recombination; ITH, intratumor heterogeneity; MMR, mismatch repair; NER, nucleotide excision repair; NHEJ, non-homologous end joining, ORR, objective response rate.

**Table S7. Kaplan-Meier survival analysis of MMR, NER, HR, FA, Checkpoint, BER, NHEJ alterations for PFS**

| **Pathological subtype** | **Total number (n)** | **Progression (n)** | **DDR altered status** | **PFS (median, days)** | **95% CI, lower** | **95% CI, upper** | **P-value** |
| --- | --- | --- | --- | --- | --- | --- | --- |
| LADC | 7 | 4 | MMR deficient type | 199 | 74.082 | 323.918 | 0.4 |
|  | 27 | 9 | MMR proficient type | 308 | 22.233 | 593.767 |  |
| LUSC | 4 | 0 | MMR deficient type | NA | NA | NA | 0.15 |
|  | 29 | 12 | MMR proficient type | NA | NA | NA |  |
| SCLC | 1 | 1 | MMR deficient type | 134 | NA | NA | **0.028** |
|  | 33 | 15 | MMR proficient type | 341 | 234.299 | 447.701 |  |
| LADC | 2 | 1 | NER deficient type | 84 | NA | NA | 0.13 |
|  | 32 | 12 | NER proficient type | 308 | 0 | 656.653 |  |
| LUSC | 4 | 3 | NER deficient type | 78 | 65.197 | 90.803 | **0.00056** |
|  | 29 | 9 | NER proficient type | NA | NA | NA |  |
| SCLC | 2 | 1 | NER deficient type | 168 | NA | NA | 0.45 |
|  | 32 | 15 | NER proficient type | 341 | 233.752 | 448.248 |  |
| LADC | 8 | 2 | HR deficient type | NA | NA | NA | 0.77 |
|  | 26 | 11 | HR proficient type | 273 | 180.008 | 365.992 |  |
| LUSC | 6 | 2 | HR deficient type | NA | NA | NA | 0.97 |
|  | 27 | 10 | HR proficient type | NA | NA | NA |  |
| SCLC | 8 | 5 | HR deficient type | 197 | 164.656 | 229.344 | **0.04** |
|  | 26 | 11 | HR proficient type | 411 | 286.087 | 535.913 |  |
| LADC | 3 | 2 | FA deficient type | 154 | NA | NA | 0.51 |
|  | 31 | 11 | FA proficient type | 308 | 210.32 | 405.68 |  |
| LUSC | 9 | 4 | FA deficient type | 139 | 24.976 | 253.024 | 0.1 |
|  | 24 | 8 | FA proficient type | NA | NA | NA |  |
| SCLC | 5 | 2 | FA deficient type | 431 | NA | NA | 0.49 |
|  | 29 | 14 | FA proficient type | 319 | 203.245 | 434.755 |  |
| LADC | 5 | 1 | Cell cycle checkpoint deficient type | NA | NA | NA | 0.42 |
|  | 29 | 12 | Cell cycle checkpoint proficient type | 273 | 178.516 | 367.484 |  |
| LUSC | 5 | 1 | Cell cycle checkpoint deficient type | NA | NA | NA | 0.76 |
|  | 28 | 11 | Cell cycle checkpoint proficient type | 289 | NA | NA |  |
| SCLC | 1 | 0 | Cell cycle checkpoint deficient type | NA | NA | NA | 0.47 |
|  | 33 | 16 | Cell cycle checkpoint proficient type | NA | NA | NA |  |
| LADC | 10 | 6 | BER deficient type | 231 | 165.219 | 296.781 | **0.026** |
|  | 24 | 7 | BER proficient type | NA | NA | NA |  |
| LUSC | 5 | 3 | BER deficient type | 78 | 0 | 171.1 | 0.097 |
|  | 28 | 9 | BER proficient type | NA | NA | NA |  |
| SCLC | 5 | 1 | BER deficient type | 411 | NA | NA | 0.31 |
|  | 29 | 15 | BER proficient type | 319 | 221.421 | 416.579 |  |
| LADC | 3 | 1 | NHEJ deficient type | NA | NA | NA | 0.36 |
|  | 31 | 12 | NHEJ proficient type | 273 | 180.787 | 365.213 |  |
| LUSC | 2 | 1 | NHEJ deficient type | 70 | NA | NA | **0.00031** |
|  | 31 | 11 | NHEJ proficient type | NA | NA | NA |  |
| SCLC | 2 | 1 | NHEJ deficient type | 397 | NA | NA | 0.97 |
|  | 32 | 15 | NHEJ proficient type | 341 | 214.7 | 467.3 |  |

Note: BER, base excision repair; DDR, DNA damage response and repair; FA, Fanconi anemia; HR, homologous recombination; ITH, intratumor heterogeneity; MMR, mismatch repair; NER, nucleotide excision repair; NHEJ, non-homologous end joining, PFS, progression-free survival; LADC, lung adenocarcinoma; LUSC, lung squamous cell carcinoma; SCLC, small cell lung cancer.

**Table S8. Kaplan-Meier survival analysis of MMR, NER, HR, FA, Checkpoint, BER, NHEJ alterations for OS**

| **Pathological subtype** | **Total number (n)** | **Dead (n)** | **DDR altered status** | **OS (median, days)** | **95% CI, lower** | **95% CI, upper** | **P-value** |
| --- | --- | --- | --- | --- | --- | --- | --- |
| LADC | 6 | 3 | MMR deficient type | 239 | NA | NA | 0.76 |
|  | 27 | 17 | MMR proficient type | 438 | 273.464 | 602.536 |  |
| LUSC | 4 | 3 | MMR deficient type | 351 | 0 | 836.1 | 0.8 |
|  | 27 | 17 | MMR proficient type | 358 | 0 | 802.416 |  |
| SCLC | 1 | 0 | MMR deficient type | NA | NA | NA | 0.27 |
|  | 25 | 19 | MMR proficient type | NA | NA | NA |  |
| LADC | 1 | 1 | NER deficient type | 239 | NA | NA | 0.39 |
|  | 32 | 19 | NER proficient type | 438 | 109.535 | 766.465 |  |
| LUSC | 4 | 4 | NER deficient type | 155 | 0 | 333.36 | **0.02** |
|  | 27 | 16 | NER proficient type | 554 | 90.237 | 1017.763 |  |
| SCLC | 2 | 2 | NER deficient type | 245 | NA | NA | 0.96 |
|  | 24 | 17 | NER proficient type | 343 | 297.391 | 388.309 |  |
| LADC | 7 | 4 | HR deficient type | 726 | 145.507 | 1306.493 | 0.48 |
|  | 26 | 16 | HR proficient type | 346 | 66.166 | 625.834 |  |
| LUSC | 7 | 5 | HR deficient type | 274 | 222.675 | 325.325 | 0.44 |
|  | 24 | 15 | HR proficient type | 543 | 197.328 | 888.672 |  |
| SCLC | 6 | 4 | HR deficient type | 582 | 460.775 | 703.225 | 0.25 |
|  | 20 | 15 | HR proficient type | 334 | 305.512 | 362.488 |  |
| LADC | 2 | 1 | FA deficient type | 213 | NA | NA | 0.86 |
|  | 31 | 19 | FA proficient type | 438 | 132.6 | 743.4 |  |
| LUSC | 9 | 7 | FA deficient type | 155 | 128.704 | 181.296 | 0.17 |
|  | 22 | 13 | FA proficient type | 543 | 86.305 | 999.695 |  |
| SCLC | 4 | 3 | FA deficient type | 334 | 0 | 796.56 | 0.94 |
|  | 22 | 16 | FA proficient type | 343 | 293.586 | 392.414 |  |
| LADC | 6 | 3 | Cell cycle checkpoint deficient type | 726 | 511.933 | 940.067 | 0.27 |
|  | 27 | 17 | Cell cycle checkpoint proficient type | 303 | 121.502 | 484.498 |  |
| LUSC | 3 | 1 | Cell cycle checkpoint deficient type | NA | NA | NA | 0.4 |
|  | 28 | 19 | Cell cycle checkpoint proficient type | 351 | 11.338 | 690.662 |  |
| SCLC | 1 | 1 | Cell cycle checkpoint deficient type | 110 | NA | NA | **0.00079** |
|  | 25 | 18 | Cell cycle checkpoint proficient type | 365 | 316.039 | 413.961 |  |
| LADC | 8 | 4 | BER deficient type | 726 | NA | NA | 0.35 |
|  | 25 | 16 | BER proficient type | 303 | 0 | 627.773 |  |
| LUSC | 5 | 3 | BER deficient type | 241 | 56.352 | 425.648 | 0.84 |
|  | 26 | 17 | BER proficient type | 358 | 79.415 | 636.585 |  |
| SCLC | 3 | 3 | BER deficient type | 365 | 307.388 | 422.612 | 0.65 |
|  | 23 | 16 | BER proficient type | 343 | 283.524 | 402.476 |  |
| LADC | 3 | 0 | NHEJ deficient type | NA | NA | NA | 0.068 |
|  | 30 | 20 | NHEJ proficient type | NA | NA | NA |  |
| LUSC | 2 | 2 | NHEJ deficient type | 59 | NA | NA | **0.025** |
|  | 29 | 18 | NHEJ proficient type | 543 | 186.104 | 899.896 |  |
| SCLC | 1 | 1 | NHEJ deficient type | 496 | NA | NA | 0.98 |
|  | 25 | 18 | NHEJ proficient type | 343 | 292.407 | 390.593 |  |

Note: BER, base excision repair; DDR, DNA damage response and repair; FA, Fanconi anemia; HR, homologous recombination; ITH, intratumor heterogeneity; MMR, mismatch repair; NER, nucleotide excision repair; NHEJ, non-homologous end joining, OS, overall survival; LADC, lung adenocarcinoma; LUSC, lung squamous cell carcinoma; SCLC, small cell lung cancer.

**Table S9. Multivariate COX regression analysis of factors with statistical significance after univariate analysis**

| Factor | HR | 95% CI | P-value |
| --- | --- | --- | --- |
| Stage (III vs. IV) | 0.278 | 0.146-0.531 | **0.000** |
| Smoking (no vs. yes) | 0.567 | 0.308-1.045 | 0.069 |
| *FANCI* mutation (no vs. yes) | 0.078 | 0.010-0.644 | **0.018** |
| *ERCC2* mutation (no vs. yes) | 16.951 | 0.322-893.673 | 0.162 |
| *ERCC4* mutation (no vs. yes) | NA | NA | NA |
| *PARP2* mutation (no vs. yes) | NA | NA | NA |
| *RAD51B* mutation (no vs. yes) | NA | NA | NA |
| *BAP1* mutation (no vs. yes) | 0.126 | 0.016-1.014 | 0.051 |
| *FANCB* mutation (no vs. yes) | 0.137 | 0.031-0.614 | **0.009** |
| *MLH3* mutation (no vs. yes) | 0.504 | 0.121-2.105 | 0.347 |

**Table S10. Mutation frequency of immune related genes**

| Gene_type | Gene_symble | The number of samples with mutations in the gene in DDR altered samples | Corresponding mutation frequency (denominator: 67) | The number of samples with mutations in the gene in DDR wild samples | Corresponding mutation frequency (denominator: 55) | Odds Ratio | P-value |
| --- | --- | --- | --- | --- | --- | --- | --- |
| Immune Inhibitors | CTLA4 | 1 | 0.015 | 0 | 0 | inf | 1 |
| Immune Inhibitors | IDO1 | 1 | 0.015 | 1 | 0.018 | 0.818 | 1 |
| Immune Inhibitors | TGFB1 | 5 | 0.075 | 3 | 0.055 | 1.398 | 0.938 |
| Immune Inhibitors | BTLA | 0 | 0 | 0 | 0 | nan | 1 |
| Immune Inhibitors | CD160 | 0 | 0 | 0 | 0 | nan | 1 |
| Immune Inhibitors | CD274 | 0 | 0 | 0 | 0 | nan | 1 |
| Immune Inhibitors | LAG3 | 1 | 0.015 | 1 | 0.018 | 0.818 | 1 |
| Immune Inhibitors | PDCD1LG2 | 0 | 0 | 0 | 0 | nan | 1 |
| Immune Inhibitors | TNFRSF14 | 0 | 0 | 0 | 0 | nan | 1 |
| Immune Inhibitors | Il10 | 0 | 0 | 0 | 0 | nan | 1 |
| Immune Inhibitors | IL10RB | 0 | 0 | 0 | 0 | nan | 1 |
| Immune Stimulator | CD80 | 0 | 0 | 0 | 0 | nan | 1 |
| Immune Stimulator | ICOSLG | 2 | 0.03 | 0 | 0 | inf | 0.501 |
| Immune Stimulator | ICOS | 0 | 0 | 0 | 0 | nan | 1 |
| Immune Stimulator | TNFSF9 | 0 | 0 | 0 | 0 | nan | 1 |
| Immune Stimulator | TNFRSF4 | 4 | 0.06 | 0 | 0 | inf | 0.183 |
| Immune Stimulator | TNFSF4 | 0 | 0 | 0 | 0 | nan | 1 |
| Immune Stimulator | CD70 | 0 | 0 | 0 | 0 | nan | 1 |
| Immune Stimulator | TNFSF18 | 1 | 0.015 | 1 | 0.018 | 0.818 | 1 |
| Immune Stimulator | IL6 | 0 | 0 | 0 | 0 | nan | 1 |
| Immune Stimulator | STING1 | 0 | 0 | 0 | 0 | nan | 1 |
| Immune Stimulator | CD27 | 0 | 0 | 0 | 0 | nan | 1 |
| Immune Stimulator | TNFRSF13B | 0 | 0 | 1 | 0.018 | 0 | 0.451 |
| Immune Stimulator | TNFRSF17 | 0 | 0 | 0 | 0 | nan | 1 |
| Immune Stimulator | IL6R | 0 | 0 | 1 | 0.018 | 0 | 0.451 |
| Immune Stimulator | CD86 | 0 | 0 | 1 | 0.018 | 0 | 0.451 |
| Immune Stimulator | CD28 | 0 | 0 | 0 | 0 | nan | 1 |
| Immune Stimulator | TNFRSF18 | 0 | 0 | 1 | 0.018 | 0 | 0.451 |
| Immune Stimulator | TNFSF13B | 0 | 0 | 0 | 0 | nan | 1 |
| Immune Stimulator | TNFRSF13C | 0 | 0 | 2 | 0.036 | 0 | 0.201 |
| Immune Stimulator | TNFSF13 | 1 | 0.015 | 0 | 0 | inf | 1 |
| Immune Stimulator | CD40LG | 2 | 0.03 | 1 | 0.018 | 1.662 | 0.862 |
| Immune Stimulator | CD40 | 0 | 0 | 2 | 0.036 | 0 | 0.201 |
| Type I IFNs | IFNA1 | 0 | 0 | 0 | 0 | nan | 1 |
| Type I IFNs | IFNA2 | 0 | 0 | 0 | 0 | nan | 1 |
| Type I IFNs | IFNA4 | 0 | 0 | 0 | 0 | nan | 1 |
| Type I IFNs | IFNA5 | 0 | 0 | 0 | 0 | nan | 1 |
| Type I IFNs | IFNA6 | 0 | 0 | 0 | 0 | nan | 1 |
| Type I IFNs | IFNA7 | 1 | 0.015 | 1 | 0.018 | 0.818 | 1 |
| Type I IFNs | IFNA8 | 1 | 0.015 | 0 | 0 | inf | 1 |
| Type I IFNs | IFNA10 | 1 | 0.015 | 0 | 0 | inf | 1 |
| Type I IFNs | IFNA13 | 1 | 0.015 | 1 | 0.018 | 0.818 | 1 |
| Type I IFNs | IFNB1 | 1 | 0.015 | 1 | 0.018 | 0.818 | 1 |
| MHC Class I | HLA-A | 1 | 0.015 | 1 | 0.018 | 0.818 | 1 |
| MHC Class I | TAP1 | 1 | 0.015 | 0 | 0 | inf | 1 |
| MHC Class I | TAP2 | 2 | 0.03 | 0 | 0 | inf | 0.501 |
| MHC Class I | HLA-B | 0 | 0 | 0 | 0 | nan | 1 |
| MHC Class I | HLA-C | 0 | 0 | 0 | 0 | nan | 1 |
| MHC Class I | B2M | 0 | 0 | 0 | 0 | nan | 1 |
| MHC Class II | HLA-DRB6 | 0 | 0 | 0 | 0 | nan | 1 |
| MHC Class II | HLA-DQB2 | 0 | 0 | 0 | 0 | nan | 1 |
| MHC Class II | HLA-DPB2 | 0 | 0 | 0 | 0 | nan | 1 |
| MHC Class II | HLA-DRB1 | 1 | 0.015 | 0 | 0 | inf | 1 |
| MHC Class II | HLA-DRB5 | 1 | 0.015 | 0 | 0 | inf | 1 |
| MHC Class II | HLA-DQA1 | 1 | 0.015 | 0 | 0 | inf | 1 |
| MHC Class II | HLA-DQA2 | 0 | 0 | 0 | 0 | nan | 1 |
| MHC Class II | HLA-DQB1 | 0 | 0 | 2 | 0.036 | 0 | 0.201 |
| MHC Class II | HLA-DPA1 | 1 | 0.015 | 0 | 0 | inf | 1 |
| MHC Class II | HLA-DPB1 | 1 | 0.015 | 0 | 0 | inf | 1 |
| MHC non-class | HLA-G | 3 | 0.045 | 0 | 0 | inf | 0.317 |
| MHC non-class | HLA-E | 1 | 0.015 | 0 | 0 | inf | 1 |
| MHC non-class | HLA-F | 0 | 0 | 0 | 0 | nan | 1 |

Note: DDR, DNA damage response and repair; IFN, interferon; MHC, major histocompatibility complex.

**Table S11. Immune-related genes between DDR-deficient and DDR-proficient samples without *EGFR* or *ALK* mutations from cBioportal database**

| LADC-TCGA | | | | | |
| --- | --- | --- | --- | --- | --- |
| **Gene** | **Entrez_ID** | **Mutant_avg_zscore** | **Wild_avg_zscore** | **logFC** | **PValue** |
| STING1 | 340061 | -0.305104255 | 0.3247884 | -0.079444446 | 0.010069935 |
| TNFRSF13B | 23495 | -0.292766901 | 0.311655088 | -0.387868294 | 0.01403861 |
| HLA-DRB6 | 3128 | -0.290241841 | 0.308967121 | -0.203284954 | 0.014388173 |
| CD28 | 940 | -0.2773536 | 0.295247381 | -0.162940622 | 0.020166533 |
| HLA-DQB2 | 3120 | -0.259645022 | 0.276396314 | -0.168040328 | 0.029469152 |
| TNFSF13 | 8741 | -0.243636507 | 0.259354992 | -0.054591967 | 0.041385057 |
| HLA-DQA2 | 3118 | -0.237841398 | 0.253186004 | -0.142089948 | 0.046680504 |
| CD70 | 970 | -0.237538906 | 0.252863996 | -0.276235378 | 0.048418253 |
| LUSC-TCGA | | | | | |
| **Gene** | **Entrez_ID** | **Mutant_avg_zscore** | **Wild_avg_zscore** | **logFC** | **PValue** |
| TNFRSF4 | 7293 | -0.234978958 | 0.328970542 | -0.155945934 | 0.017014227 |
| TGFB1 | 7040 | -0.195213081 | 0.273298313 | -0.047917784 | 0.033003974 |
| SCLC_ucologene_2015 | | | | | |
| **Gene** | **Entrez_ID** | **Mutant_avg_zscore** | **Wild_avg_zscore** | **logFC** | **PValue** |
| CD40 | 958 | 0.310907974 | -0.260295048 | 0.325717517 | 0.011650981 |
| TNFRSF14 | 8764 | 0.282313425 | -0.236355425 | 0.154108608 | 0.021553577 |

Note: DDR, DNA damage response and repair; LADC, lung adenocarcinoma; LUSC, lung squamous cell carcinoma; SCLC, small cell lung cancer; TCGA, The Cancer Genome Atlas.

| **Table S12. Infiltrated immune cells between DDR-deficient and DDR-proficient samples without *EGFR* or *ALK* mutations from cBioportal database** | | | | | |
| --- | --- | --- | --- | --- | --- |
| LADC-TCGA | | | | | |
| **Cell_type** | | **Mutant_avg_cellfraction** | **Wild_avg_cellfraction** | **logFC** | **PValue** |
| Mast cells resting | | 0.035892951 | 0.067638884 | -0.914152285 | 0.003251975 |
| B cells memory | | 0.008456184 | 0.019388122 | -1.197094342 | 0.025338986 |
| Dendritic cells resting | | 0.007294347 | 0.017881203 | -1.29359297 | 0.034659952 |
| Mast cells activated | | 0.021377635 | 0.007817499 | 1.451323153 | 0.043838075 |
| LUSC-TCGA | | | | | |
| **Cell_type** | **Mutant_avg_cellfraction** | | **Wild_avg_cellfraction** | **logFC** | **PValue** |
| Macrophages M2 | 0.224613178 | | 0.175145039 | 0.358892446 | 0.004418113 |
| Macrophages M0 | 0.140482273 | | 0.183216648 | -0.383162511 | 0.049097499 |
| SCLC_ucologene_2015 | | | | | |
| **Cell_type** | **Mutant_avg_cellfraction** | | **Wild_avg_cellfraction** | **logFC** | **PValue** |

Note: DDR, DNA damage response and repair; LADC, lung adenocarcinoma; LUSC, lung squamous cell carcinoma; SCLC, small cell lung cancer; TCGA, The Cancer Genome Atlas.
